# Leveraging Network-Based Transcriptome Analysis from Mouse Tumor Models and Explainable Artificial Intelligence to Advance the Understanding of the Antitumor Activity of Lenvatinib

**DOI:** 10.3390/cancers18071067

**Published:** 2026-03-25

**Authors:** Haruna Imamura, Sufeng Chiang, Megumi Kuronishi, Yasuhiro Funahashi, Taiko Nishino, Ayako Yachie

**Affiliations:** 1 The Systems Biology Institute, Saisei Ikedayama Bldg. 5-10-25 Higashi Gotanda, Shinagawa 141-0022, Tokyo, Japan; imamura@sbi.jp (H.I.);; 2 Tsukuba Research Laboratories, Eisai Co., Ltd., 5-1-3 Tokodai, Tsukuba 300-2635, Ibaraki, Japany-funahashi@hhc.eisai.co.jp (Y.F.)

**Keywords:** lenvatinib, tumor microenvironment, drug response, network-based transcriptome analysis, machine learning, explainable AI

## Abstract

Background/Objectives: Understanding the mechanisms of drug response plays an essential role in predicting effects prior to drug administration and advancing personalized medicine by optimizing treatment strategies. This study aimed to identify gene combinations that can predict the antitumor activity of lenvatinib, which is a multi-targeted tyrosine kinase inhibitor. Methods: Cancer- and drug-response-related gene sets were identified by mapping gene expression profiles of previously reported syngeneic mouse tumor models onto a protein–protein interaction network and extracting subnetworks comprising nodes where high expression levels were clustered. The scores for these network modules were calculated using the expression data of mouse tumor models prior to drug administration. These scores were used to train a machine learning (ML) model of drug response to lenvatinib by narrowing down the parameter space using hepatocellular carcinoma patient-derived xenograft (HCC PDX) models acquired in this study. Results: Using this integrative framework, we identified several network modules including those involved in the nerve growth factor signaling pathway, Wnt signaling pathway, and interleukin signaling pathways, that were consistently prioritized as informative features across PDX models and human patient data from The Cancer Genome Atlas. Conclusions: These network modules exhibit biological functions that are linked to the known targets of lenvatinib in the cancer cells or the tumor microenvironment, highlighting their potential relevance as determinants of drug response.

## Simple Summary

This study leveraged network-based transcriptomic characteristics derived from mouse models combined with an explainable machine learning framework to identify specific gene network modules that predict the effectiveness of lenvatinib. The findings of the present study underscore the potential of network-informed, data-driven models for advancing precision oncology.

## 1. Introduction

Lenvatinib is a multi-targeted tyrosine kinase inhibitor that targets vascular endothelial growth factor receptor (VEGFR) 1–3, platelet-derived growth factor receptor α (PDGFRα), fibroblast growth factor receptor (FGFR) 1–4, KIT and RET proto-oncogenes [1,2,3]. Lenvatinib monotherapy has been used for the treatment of differentiated thyroid cancer refractory to radioactive-iodine, unresectable thyroid cancer [4,5,6], and unresectable hepatocellular carcinoma (HCC) [6,7,8,9]. Notably, a combination of lenvatinib and pembrolizumab, a humanized monoclonal anti-programmed cell death-1 (PD-1) antibody, has been used for the management of advanced endometrial carcinoma exhibiting disease progression during or after systemic therapy in patients who are ineligible for curative surgery or radiation [9,10]. Lenvatinib + pembrolizumab is the first-line treatment for adult patients with advanced renal cell carcinoma (RCC). Lenvatinib + everolimus, a mammalian target of rapamycin (mTOR) kinase inhibitor, is approved for the treatment of adult patients with advanced RCC who have previously received anti-angiogenic therapy [9,11].

The analysis of genome-wide expression profiles has been widely used to identify genes related to drug treatment [9,10,11,12,13,14]. This technique involves the selection of markers according to the score assigned to each gene for the ability to determine the response to the drug based on its expression pattern. Numerous biomarkers that can predict the response to immune checkpoint inhibitors have been identified in previous genomic and transcriptomic studies [15,16,17,18,19]. However, biological heterogeneity, such as cellular variation within the tissues or genetic variation across patients, and technical fluctuations in the measurements, are challenges associated with expression-based classification. The appearance of “noise” in the gene expression profiles may weaken the discriminative power of individual genes [20,21]. Biological processes often affect several genes simultaneously through direct or indirect interactions to function in coordination. Such groupings constitute protein complexes, signaling cascades, or higher-order cellular processes and are sometimes known as “functional modules.” The composition of gene expression in such functional modules is more robust against the “noise” present in the expression profiles from the biological and statistical point of view. Several studies have suggested combining gene expression measurements over groups of genes in functional modules, rather than using the expression levels of individual genes [21,22,23,24,25]. Building on this concept, diffusion-based network approaches have emerged as powerful tools for identifying such functional modules by integrating gene-level signals with molecular interaction networks. These methods enable the extraction of biologically coherent subnetworks from diverse quantitative measurements. Recent studies have applied network propagation to transcriptomic profiles, demonstrating that diffusion frameworks provide a general mechanism for amplifying coordinated biological signals in heterogeneous omics data [26,27].

A tumor is an ecosystem wherein cancer cells and diverse non-malignant cells of the tumor microenvironment (TME) interact with each other [28]. The interactions with cancer and other cells within the TME shape and modify the transcriptome and phenotype of non-malignant components, including blood vessels and immune cells. Blood vessels facilitate the growth and metastasis of cancer cells by supplying them with essential nutrients and oxygen. Immune cells such as T cells, macrophages, and dendritic cells are present within the TME. Individual cell lineages play various roles in antitumor immunity, including the elimination of cancer cells or suppression of the antitumor immune environment to facilitate cancer progression. The interaction between non-malignant and cancerous cells plays an important role in the processes of tumor development, disease progression, and drug resistance [29,30]. Lenvatinib, an angiogenesis inhibitor, has exhibited broad-spectrum antitumor activity in human tumor xenograft models. Consequently, previous studies have examined the association of lenvatinib with microvascular density and pericyte coverage of tumor vasculature, which are factors correlated with its antitumor activity [3]. The antitumor activity of lenvatinib is associated with a reduction in the number of tumor-associated macrophages (TAMs) in the TME. Notably, this activity was attenuated under conditions characterized by CD8^+^ T cell depletion, suggesting its role as an immunomodulator [31]. Identification of the changes in the expression in the TME and their association with antitumor activity would deepen our understanding of drug activity in individual patients. Thus, the identification of new sets of genes beyond the limitation of existing knowledge is essential for achieving a more comprehensive view of the “functional modules” associated with lenvatinib response in TME. However, the identification of pathway-based signatures of the lenvatinib response at the transcriptome level is conceptually and technically challenging owing to the absence of large-scale data regarding gene expression and the antitumor activity of lenvatinib.

A recent study has reported the RNA-sequencing (RNA-seq) data of 12 syngeneic tumor mouse models treated with lenvatinib [32]. A new methodology to define gene sets that are biologically relevant to drug effects at the transcriptome level was developed using mouse datasets in the present study. “Non-canonical network modules” were defined using a method based on a network diffusion algorithm that integrates the changes in gene expression across connected nodes in a molecular network. Machine learning (ML) techniques were applied subsequently to explore the associations of the antitumor effect of lenvatinib with the non-canonical network modules. Interpretable ML models were constructed by training the RNA-seq dataset of syngeneic mouse models and testing it with the RNA-seq data of HCC patient-derived xenograft (PDX) models. These ML models were used to identify network modules that contributed to predicting lenvatinib response. The key network modules in drug effects were identified by applying ML models to HCC PDX model data and liver hepatocellular carcinoma (LIHC) patient data from the Cancer Genome Atlas (TCGA). Some of these have functions which are associated with lenvatinib, such as angiogenesis and immune system modulation, whereas others are novel candidates for future studies.

## 2. Results

### 2.1. Syngeneic Mouse Tumor Models Classified Based on the Antitumor Activity of Lenvatinib

Syngeneic mouse are immunocompetent mice inoculated with mouse cancer cells that reflect the complexity of the interaction between the cancer and non-malignant cells in the TME. The immune system of the host mouse recognizes tumor cells as its own and reflects the complexity of tumor development and the response to treatment. Accordingly, these models have been used to examine antitumor response following treatment with lenvatinib in an immunocompetent tumor microenvironment.

**Figure 1:**
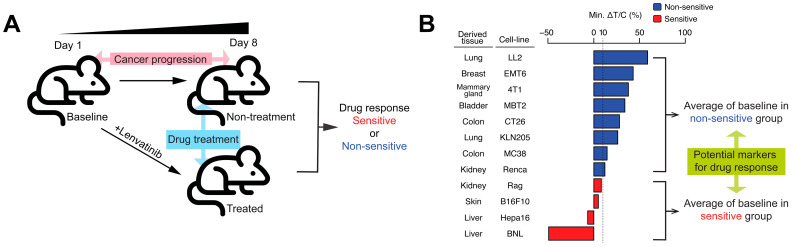
Syngeneic mouse tumor models were classified based on the antitumor activity of lenvatinib. (**A**) Experimental scheme using syngeneic mouse models to evaluate the antitumor effect of lenvatinib. (**B**) Classification of the sensitive and non-sensitive models based on lenvatinib antitumor activity. The color in the bar chart indicates the classification as sensitive (red) or non-sensitive (blue) based on the minimum Δ*T*/*C* in the time series with 10% as the threshold (dashed line).

RNA-seq data and the antitumor effects of lenvatinib in 12 syngeneic mouse models were reported in a previous study [32]. RNA-seq data were obtained from the inoculated tumors during the early and late stages of tumor development and drug administration. The overall experimental framework is summarized in Figure 1A.

Lenvatinib exerted a range of antitumor effects across a panel of 12 syngeneic mouse models. The mouse models were divided into lenvatinib “sensitive” or relatively resistant “non-sensitive” models for further binary classification framework based on the antitumor effect evaluated according to the tumor growth suppression and the tumor volume reduction caused by lenvatinib (Figure 1B; see Section 4.1).

To assess the robustness of this dichotomization, we examined how different Δ*T*/*C* thresholds affected the classification of the 12 models. A 15% cutoff produced a balanced distribution (6 sensitive vs. 6 non-sensitive), but this threshold did not align with the biological distinction recognized by the in vivo experimental team. In contrast, the 10% threshold represented the smallest cutoff that yielded a reasonably balanced classification (4 vs. 8) while remaining consistent with long-standing empirical observations of clear lenvatinib responsiveness. Therefore, we adopted the 10% Δ*T*/*C* threshold as the final definition of lenvatinib sensitivity in all subsequent analyses. We further assessed the robustness of this phenotype dichotomization against modest uncertainty in Δ*T*/*C*-based classification. Sensitivity analyses using alternative Δ*T*/*C* thresholds and label perturbations indicated that downstream network-level analyses were stable with respect to this threshold choice (see Section S5 in Supplementary Material for further details).

### 2.2. Extraction of Non-Canonical Network Modules by Network Diffusion

The identification of the changes in the expression related to cancer progression or drug administration, and understanding their relationship with antitumor activity, are critical for evaluating drug effects at the transcriptome level. To characterize the cancer cells and TME from multiple perspectives, we considered factors associated with cancer progression, treatment-induced changes, and potential markers of drug response.

Based on these considerations, three biological dimensions were defined for identifying novel functional modules, namely “cancer progression,” “drug treatment,” and “drug response.” RNA-seq data were obtained from tumors under multiple conditions (Figure 1A: baseline, treated, and non-treatment). In addition, lenvatinib sensitivity status (sensitive vs. non-sensitive), as defined based on antitumor activity, was incorporated into this conceptual framework (Figure 1B).

**Figure 2:**
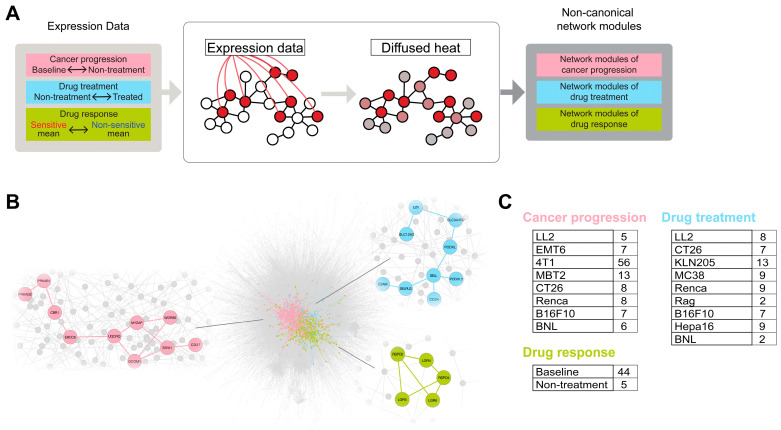
Identification of the non-canonical network modules based on network diffusion. (**A**) Schematic of the pipeline for identifying network modules from the expression data of syngeneic mouse tumor models using the network diffusion method. (**B**) Schematic diagram of the network modules derived from a reference PPI network. (**C**) Number of network modules obtained from each dataset.

To identify “non-canonical” functional network modules corresponding to these biological dimensions, a network-diffusion-oriented strategy designed to detect high-weight subnetworks within a weighted network was adopted [33]. In this approach, gene-level metrics derived from RNA-seq data were assigned as node weights on a protein–protein interaction (PPI) network. These node weights were subsequently propagated to neighboring genes along network edges according to the underlying network topology, such that signal from highly weighted nodes was propagated to their network neighbors (Figure 2A). This diffusion process allows locally connected genes to collectively reflect transcriptional changes, enabling the extraction of densely connected subnetworks with elevated signal. Using this approach, cancer- or TME-related “non-canonical” functional network modules were identified from the PPI network (Figure 2B,C; see Section 4.6).

Accordingly, network modules were identified to represent each biological dimension as follows. The differences in expression between “baseline” and “non-treatment” were used for defining the network modules of “cancer progression” (Figure 2, pink). The resulting modules can be considered gene subsets that exhibit the changes in the expression levels during cancer progression. The differences in expression between “treatment” and “non-treatment” were used for defining the network modules of “drug treatment” (Figure 2, blue). The resulting modules were interpreted as gene subsets exhibiting the changes in expression following treatment with lenvatinib. The differences between the “sensitive” and “non-sensitive” groups of “baseline” or “non-treatment” were used for defining the network modules of “drug response” (Figure 2, green). These results indicated potential markers of drug responsiveness that can be observed prior to lenvatinib administration.

Network modules with overlapping members were merged to create a final list (Table S1). The network modules of “cancer progression,” “drug treatment,” and “drug response” included 101 (Table S1A), 59 (Table S1B), and 46 (Table S1C) network modules, respectively. The network modules in these lists included genes related to angiogenesis [34,35,36] a biological function associated with lenvatinib; genes related to T-cell inflammation [16]; and T-cell or tumor-associated macrophage phenotype markers from a study of clear cell renal cell carcinoma [37].

### 2.3. Non-Canonical Network Modules Can Stratify the Response of the HCC PDX Models

**Figure 3:**
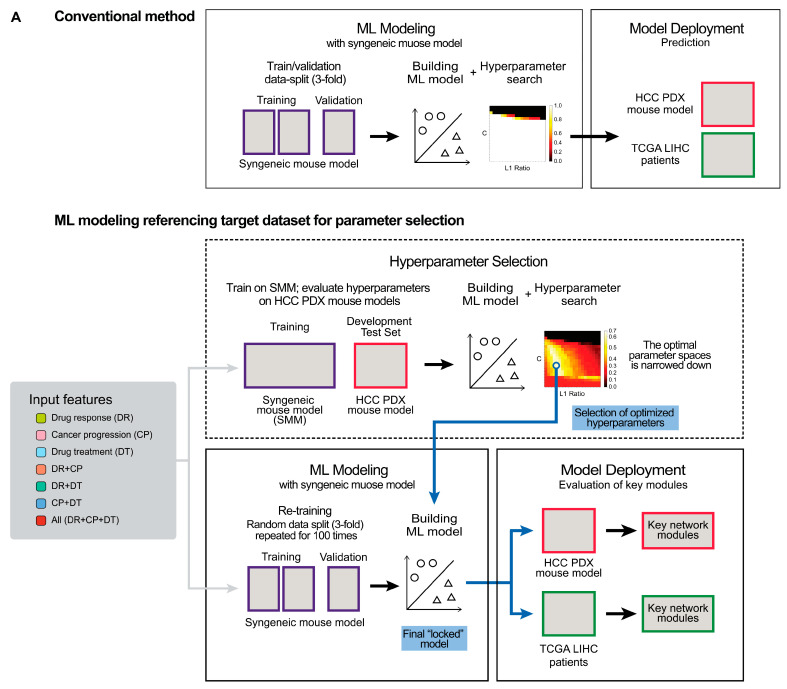

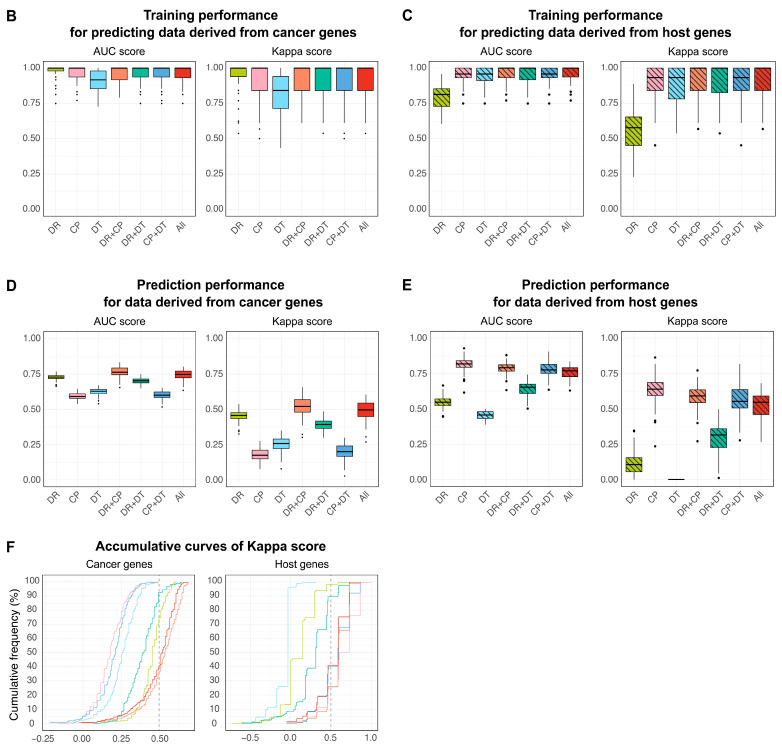
Construction and evaluation of the machine learning models. (**A**) Flowchart of the construction and evaluation of the machine learning models. (**B**) Training performance for the cancer genes in the tumors from the HCC PDX models. (**C**) Training performance for the host genes in the tumors from the HCC PDX models. (**D**) Prediction performance for the cancer genes in the tumors from the HCC PDX models. (**E**) Prediction performance for the host genes in the tumors from the HCC PDX models. (**F**) Accumulative curves of kappa scores for prediction performance. (**B**,**C**) illustrate the training performance, and (**D**,**E**) present the prediction performance for cancer or host genes (left panels: AUC score; right panels: kappa score). Boxplots were drawn using the average values of each three-fold split experiment (100 data points). (**F**) shows the cumulative curves for the kappa score of prediction performance. Three hundred data points from all the experiments (three-fold split for 100 iterations) were used. A kappa score of 0.5 (shown as a dashed line) was used as the cutoff to define the predictive ML models (Table S3).

Supervised ML analyses were performed on 12 syngeneic mouse tumor datasets to develop a predictive modeling framework for lenvatinib responses based on mouse models and to investigate the mechanism underlying the antitumor effects of lenvatinib (Figure 3A; see Section 4.9). The RNA-seq data collected from the syngeneic mouse models prior to lenvatinib treatment were used to compute scores for the non-canonical network modules (see Section 4.7). These scores of the syngeneic mouse subsequently used as input features for training a machine learning model to predict drug response. The mouse-derived features were used as inputs for interpretable sensitive/non-sensitive classification models, with the aim of identifying latent transcriptional factors associated with lenvatinib antitumor activity in additional mouse models and TCGA datasets.

ML models were constructed separately on the three described non-canonical network modules (single views), pairwise combination of views, and the combination of all three views with four supervised ML classifiers to evaluate the involvement of the non-canonical modules derived from cancer progression, drug response potential, or drug treatment in the prediction of the antitumor effects of lenvatinib.

The RNA-seq data of the HCC PDX models (Table S2) was used only as an external development test set to compare model performance across different hyperparameter configurations. Importantly, the HCC PDX dataset was never used for model training. All machine learning models for hyperparameter optimization were trained exclusively on the syngeneic mouse dataset, and their performance was then evaluated on the HCC PDX samples based on the observed antitumor activity (Figure S1). After selecting the hyperparameter set that yielded the best performance, a final “locked” model was retrained using only the syngeneic mouse data and subsequently applied to the HCC PDX dataset for prediction.

The RNA-seq data of the tumors from the HCC PDX models comprised a mixture of reads originating from the human liver cancer cells and mouse host cells, which formed a TME that mimics the original liver tumors. Based on the alignment of the reads to the human and mouse genome sequences, these were divided into human and mouse parts. The human gene dataset in the HCC PDX models was used to gain insights from the patient liver cancer cells (hereafter referred to as “cancer genes”). The mouse gene dataset was used to gain insights from the host cells in TME (hereafter referred to as “host genes”).

The best-performing ML classifiers and their optimal hyperparameters based on every view were determined to identify the datasets of cancer or host genes in the tumors from the HCC PDX models (see Section S2 in Supplementary Material for further details). These datasets were used to evaluate the performance of the non-canonical network modules in terms of the stratification of the lenvatinib response by building 300 ML models produced through 100 iterations of three-fold cross-validation on the syngeneic mouse data with random initial seeds.

The ML model exhibited consistently high training performance for both datasets from the cancer and host genes in the tumors obtained from the HCC PDX models, with median kappa scores of >0.75, except for the “drug response” (DR) single view ML models for the host genes (Figure 3B,C). The prediction performance showed substantial variability depending on the sets of network modules used as input features (Figure 3D,E).

In addition to AUC and Kappa score shown in Figure 3D,E, we evaluated accuracy, precision, and F1 score and statistically compared the seven feature views using one-way ANOVA followed by Tukey’s HSD tests. Significant pairwise differences were identified (see Section S3 in Supplementary Material for further details).

For ML models for the cancer genes in the tumors from the HCC PDX models, the “drug response” (DR) single view outperformed the “cancer progression” (CP) and “drug treatment” (DT) single views (Figure 3D; adjusted *p* < 0.001 across all metrics). The combination view of “drug response” and “cancer progression” (DR + CP) further improved performance compared to the “drug response” (DR) single view, whereas the combination of “drug response” and “drug treatment” (DR + DT) resulted in a significant decrease in performance (adjusted *p* < 0.001 across all metrics). The addition of the “drug treatment” (DT) view to the combination of “drug response” and “cancer progression” (DR + CP) sets (the All view) significantly reduced prediction performance (adjusted *p* < 0.01 across AUC, Kappa, and F1 scores).

For the prediction of the host genes in the tumors from the HCC PDX models, the “cancer progression” (CP) single view achieved the best performance (Figure 3E), significantly outperforming all other feature views (adjusted *p* < 0.01 across AUC, Kappa, accuracy and F1 score). Combining the “drug response” set, the “drug treatment” set, or both with the “cancer progression” set (DR + CP, CP + DT, All) did not yield additional improvement; instead, these combinations resulted in significantly reduced performance compared with the CP single view (adjusted *p* < 0.01 across AUC, Kappa, accuracy and F1 score).

Several studies using gene sets identified based on canonical knowledge as inputs for ML models to predict drug responses since pathway-level features have reported better discriminative power for the prediction of cancer therapy responses. PROGENy pathways [38] and DoRothEA transcription factor (TF) regulons [39] can determine latent markers to stratify responses to immune checkpoint inhibitors [40], as detailed in the Methods (see Section 4.8). The performance of these modules was compared with that of the non-canonical network modules in predicting the lenvatinib response in the HCC PDX models. However, the pathway activity and transcription factor activity-based scores could not be tailored to discriminate the lenvatinib response of the HCC PDX models (Figure S2). This difference was statistically significant based on one-way ANOVA followed by Tukey’s HSD tests (adjusted *p* < 0.001 across AUC, Kappa, accuracy, precision and F1 scores; see Section S3 in Supplementary Material for further details).

### 2.4. Distinct Non-Canonical Network Module Patterns Specify the ML Models of the Cancer and Host Genes in the Tumors from the HCC PDX Models

The SHapley Additive exPlanations (SHAP) analysis was performed to identify the important non-canonical network modules as the key contributors in stratifying the response to lenvatinib for the ML models optimized to HCC PDX model prediction (see Section S4 in Supplementary Material for further details).

ML models deemed to have sufficiently high predictive performance were selected as “predictive” ML model from the 300 ML models (100 iterations of three-fold data split) using a kappa score of >0.5 as a threshold (Figure 3F). The views exhibiting an overall higher predictive performance among the different views used for training tended to have a greater number of predictive ML models (Table S3). The predictive ML models were subsequently subjected to SHAP-based interpretation to identify non-canonical network modules contributing to drug response classification. For this analysis, only views among the seven views with more than 50 predictive ML models were included. We selected multiple views rather than the single best one in order to examine how the contributions of non-canonical network modules varied depending on the input features. These included the “drug response” single view, the combination view of “drug response” + “cancer progression,” and the combination of all three module sets (“all”) for the cancer genes in the tumors from the HCC PDX models. The “cancer progression” single view, the combination view of “drug response” + “cancer progression,” the combination view of “cancer progression” + “drug treatment,” and the combination of all three module sets (“all”) were matched for the host genes in the tumors from the HCC PDX models.

The ranking of non-canonical network modules in SHAP analysis for predicting the effects of lenvatinib was used to infer their importance. The key network modules were defined as those with an average ranking of less than 15 across the predictive ML models (Table S4). Modules classified as Class I (average rank < 6) are considered highly informative within a given analysis setting, whereas Class II modules (6 ≤ average rank < 15) represent moderately informative features.

Most of the important non-canonical network modules for predicting the drug response with cancer genes in the HCC PDX models were obtained from the “drug response” view. In contrast, almost all the important network modules for those of the host genes were obtained from the “cancer progression” view. Only two overlaps (“drug response (baseline) N34” and “cancer progression (4T1) N48”) were observed between the important modules of the cancer genes and the host genes in the tumors from the HCC PDX models (Table S4). This finding indicates the presence of distinct non-canonical network module patterns for predicting lenvatinib responses based on the gene expression profiles of human liver cancer cells or the surrounding host environment. Notably, the addition of “drug treatment” network modules to the respective best single view (“drug response” and “cancer progression” view for cancer and host genes, respectively) as ML model inputs worsened the predictive performance (Figure 3D,F). Two “drug treatment” modules were identified as important (“drug treatment (BNL) N2” and “drug treatment (KLN205) N9” for the prediction of cancer and host genes in the HCC PDX mouse models, respectively). However, consistent with the results of ML model performance, the addition of “drug treatment” network modules did not enhance the biological insight obtained from interpreting the ML models. This observation suggests that transcriptional states present prior to treatment may carry stronger information for stratifying lenvatinib response than treatment-induced expression changes within the current modeling framework.

### 2.5. Exploratory Projection of Mouse-Derived Network Modules onto TCGA LIHC

Given that the predictive ML models for lenvatinib responses were constructed using mouse models, and that the important network modules for classification were also identified through further mouse model-based evaluations, we aimed to assess their relevance and translational potential in a human context. TCGA [41,42] provides a large set of genomic data derived from patient tumor samples, including RNA-seq expression profiles. To explore whether network modules identified from mouse models capture biologically meaningful transcriptional variation in human tumors, we projected these mouse-derived network modules onto TCGA LIHC transcriptomic data (Figure 3A). TCGA LIHC samples were grouped into model-consistent partitions based on similarity in mouse-derived network module activity profiles, as direct information on lenvatinib antitumor activity is not available in TCGA (see Section 4.10 for details of data processing and proxy label definition).

Table S4 provides a comprehensive list of candidate non-canonical network modules that were identified as informative in either the HCC PDX models or the TCGA LIHC transcriptomic analysis. From this candidate set, network modules that were consistently identified as important in both the mouse-derived HCC PDX models (cancer or host gene expression) and the TCGA-LIHC dataset were selected and summarized in Table 1. Table 1 highlights these overlapping modules and annotates whether the cross-dataset consistency was observed in cancer cells or host cells in the tumor from the HCC PDX models, together with the corresponding importance class (Class I or II) defined based on the class shared between the HCC PDX models and the TCGA LIHC analysis.

The network modules consistently identified as informative in both HCC PDX models (cancer cell or host cell gene expression) and TCGA LIHC transcriptomic data, selected from the candidate modules listed in Table S4. Modules are categorized into Class I (average rank < 6) and Class II (6 ≤ average rank < 15) based on the class commonly assigned in both the HCC PDX models and the TCGA LIHC analysis; when the assigned classes differed between datasets, the module was classified as Class II.

To quantitatively assess whether this overlap exceeded random expectation, we performed hypergeometric tests on the overlap of highly ranked network modules (average ranking < 15) between the HCC PDX models and TCGA LIHC across all comparable ML input views. After correction for multiple testing, significant enrichment of overlapping modules was observed in several views (FDR *q* < 0.05), supporting non-random cross-dataset consistency (see Section S6 in Supplementary Material for details). All network modules summarized in Table 1 were included in at least one comparison showing significant enrichment after multiple testing correction (FDR *q* < 0.05).

Collectively, these results indicate that network modules prioritized in the HCC PDX models, for both cancer and host genes expression, reflect major axes of transcriptional variation across TCGA LIHC samples. The network modules defined by the predictive ML models for lenvatinib responses, originally developed using mouse models, showed consistent relevance in capturing transcriptional variation in an independent human LIHC cohort.

### 2.6. Drug Response Network Modules Are the Main Factors in Stratifying the Lenvatinib Response of Cancer Genes in the Tumors from the HCC PDX Models

In addition to the one important module from the “drug treatment” set, 15 “drug response” modules and four “cancer progression” modules were identified as important in the predictive models for cancer genes in the tumors from the HCC PDX models. Nine important “drug response” modules (drug response (baseline) N2, N8, N19, N28, N34, N38, N40, N42, and (non-treatment) N1) and one important “cancer progression” module (cancer progression (4T1) N17) overlapped with the important network modules for the LIHC patient data from TCGA (Table 1 and Table S4). The overlap between the important network modules of the predictive models for cancer genes in the tumors from the HCC PDX models and the TCGA LIHC dataset indicates that they could be involved in the mechanism underlying the effects of lenvatinib observed in the patients. Moreover, all overlapping important network modules, except one, were derived from the “drug response” module set. Thus, the “drug response” network modules dominated the stratification of lenvatinib response in the TCGA samples.

### 2.7. Cancer Progression Network Modules Are Key Determinants of the Lenvatinib Response of Host Genes in the Tumors from the HCC PDX Models

Nine “cancer progression” modules and one “drug response” module were identified as important for the prediction of lenvatinib antitumor efficacy for host genes in the tumors from the HCC PDX models. This finding indicates that the “cancer progression” network modules dominated the predictive models of the host genes in the tumors from the HCC PDX model data. Five cancer progression modules (cancer progression (4T1) N18, N32, N50, (BNL) N3, and (EMT6) N5) and one drug response module (drug response (baseline) N34) overlapped with the important network modules for LIHC patient data from TCGA (Table 1 and Table S4). Further evaluation of the overlap between the important network modules of the predictive models for host genes in the tumors from the HCC PDX model data and the TCGA LIHC data might be useful to elucidate the association between the TME and lenvatinib responses in clinical samples.

### 2.8. Important Non-Canonical Network Modules Implicate the Mechanism Underlying Lenvatinib Antitumor Effects

**Figure 4:**
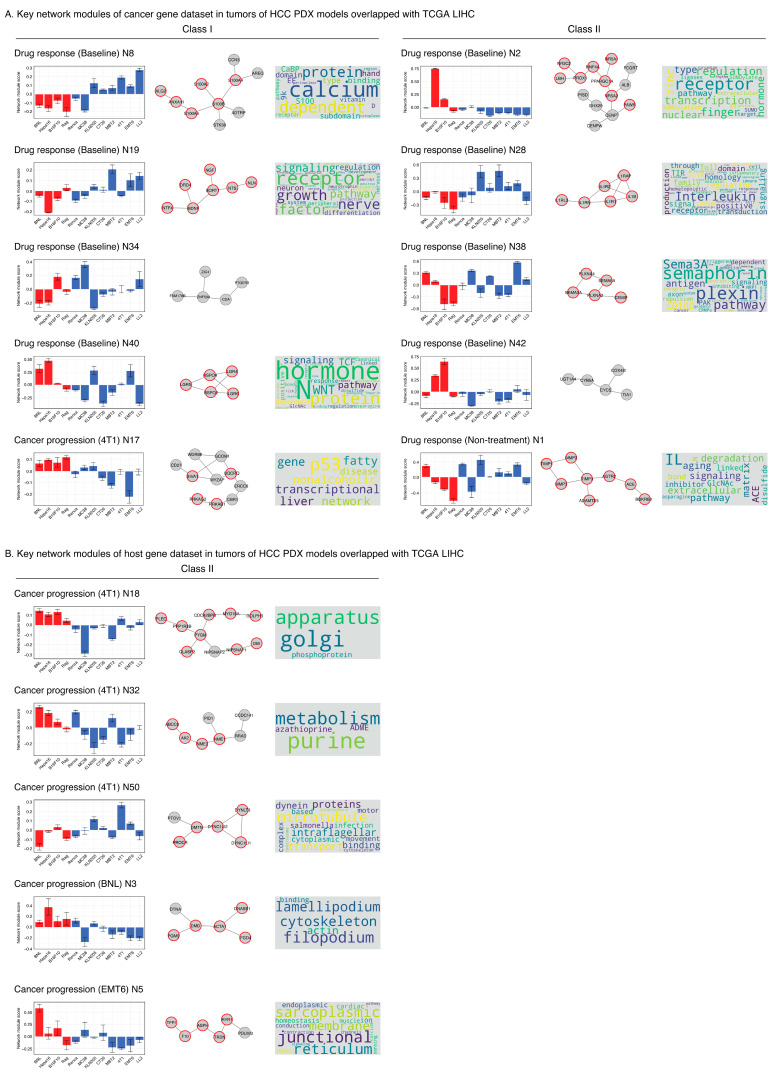
Biological functions of key network modules for predicting lenvatinib response. Each panel comprises three parts: (1) bar charts of network module scores of syngeneic mouse models from left to right in descending order of antitumor effect in Figure 1B (red: sensitive, blue: non-sensitive), (2) the network topology diagram that shows the connection of genes in the reference protein–protein interaction network with marked genes included in any of the enrichment terms in red, and (3) word cloud image of the enriched terms in Table S5. The word size reflects the relative frequency of each term. Colors are used for visual distinction only and do not represent additional quantitative information.

The connections between the genes in the PPI network and their associations were analyzed using functional term enrichment analysis to investigate the functional relevance of the network modules listed in Table 1 (Figure 4A,B, Table S5; see Section 4.11). The network module scores from the syngeneic mouse models, which were used to train the predictive ML model, were arranged in the order of their antitumor activity (Figure 4A,B). Some results exhibited a mild positive or negative correlation between the antitumor effects and scores.

The analysis of cancer genes from the tumor in the HCC PDX models was expected to reflect transcriptomic snapshots of the biological conditions in human liver cancer cells. Some of the important network modules for predicting drug responses to cancer genes in the HCC PDX models were associated with angiogenesis as well as several cancer-related pathways, including the nerve growth factor (NGF), Wnt, and interleukin signaling pathways (Figure 4A). The “drug response (baseline) N8” module includes multiple proteins belonging to the S100A family of proteins (S100A2, S100A4, S100A6). The “drug response (baseline) N19” modules include NGF as its member, as well as other members (NTF4, BDNF, SORT1), that are involved in the development of the nervous system and the nerve growth signaling pathway as well as “MAPK signaling (WIKIPATHWAY)” and “Ras signaling pathways (KEGG).” The “drug response (baseline) N38” modules include two proteins (SEMA3A and SEMA6A) from the Semaphorins (SEMA) family. Enrichment was observed in the “semaphorin-plexin signaling pathway (GO BP)” and “NRP1-triggered signaling pathways in pancreatic cancer (WIKIPATHWAYS).” The “drug response (baseline) N40” module was enriched in the “Wnt signaling pathway (KEGG).” The “drug response (baseline) N28” and “drug response (non-treatment) N1” modules were involved in the signaling pathways of interleukins (ILs). The “cancer progression (4T1) N17” module was involved in “p53 transcriptional gene network (WIKIPATHWAYS)” and “nonalcoholic liver disease (WIKIPATHWAYS).” The “drug response (baseline) N2” module was involved in terms such as “transcription regulation (UP_KW_BIOLOGICAL_PROCESS)” and multiple SUMOylation-related terms. No significantly enriched functional term was identified for “drug response (baseline) N34” and “drug response (baseline) N42” in the present analysis, indicating that their relevant function has not been unrevealed in the queried public databases yet.

The interpretation gained from host genes was expected to mirror the characteristics of the host cells in TME. The important non-canonical network modules identified by host genes in the tumors from the HCC PDX models were primarily involved in cytoskeletal cell function and cell movement (Figure 4B). The “cancer progression (BNL) N3” is involved in the cytoskeleton, filopodia, and lamellipodia. The “cancer progression (4T1) N50” module was involved in the cytoskeleton, motor protein, and microtubule-based movement. The “cancer progression (EMT6) N5” module was involved in cardiac conduction and muscle contraction. The other two important modules identified from the host genes, the “cancer progression (4T1) N18” module and the “cancer progression (4T1) N32” module, were involved in the Golgi apparatus and purine metabolism/azathioprine ADME, respectively.

These results suggest that the newly developed ML framework facilitates the identification of key network modules that function in cancer or the TME, potentially contributing to the determination of sensitivity to lenvatinib treatment. Importantly, sensitivity analyses demonstrated that the prioritization of key response-associated network modules identified here was robust to modest uncertainty in phenotype classification, including representative “drug response” modules such as “drug response (baseline) N8” module, including S100A family proteins, “drug response (baseline) N19” module, enriched for NGF signaling pathway, and “drug response (baseline) N40” module, associated with Wnt signaling pathway (see Section S5 in Supplementary Material for further details).

## 3. Discussion

### 3.1. Overview of the Framework and Key Findings

The first framework to stratify lenvatinib responses using RNA-seq data was established in the present study by integrating two available mouse model datasets. This enabled the identification of key network modules that can predict the antitumor efficacy of lenvatinib through the application of ML models to HCC PDX mouse models and TCGA patient datasets. The findings of the present study provide essential insights into the biological mechanisms underlying the effects of lenvatinib by leveraging a limited but valuable set of mouse data. Thus, the components of these network modules could serve as targets for further investigations into the response mechanisms of lenvatinib.

The tumors from the HCC PDX models comprised a mixture of human cancer cells derived from patient tumors and host mouse cells. The human gene dataset represented the characteristics of patient liver cancer cells, whereas the mouse gene dataset reflected the host cells in TME. The key network modules found in the prediction for the cancer cells of the tumors from the HCC PDX models included elements that indicate indirect or direct links to the mechanism of action of lenvatinib as well as cancer-related pathways.

### 3.2. Angiogenesis, Immune Modulation, and Wnt Signaling as Candidate Mechanisms

Several network modules are involved in the regulation of angiogenesis. The “drug response (baseline) N8” module included S100A4, a protein with S100/CaBP-9k-type calcium-binding domain. CaBP-9k is up-regulated by the estrogenicity of 2-methoxyestradiol (2-ME), a potential novel antitumor agent as an anti-angiogenic and anti-proliferative molecule [43].

NGF in the “drug response (baseline) N19” module is a novel angiogenic molecule that exerts various effects on the endothelial cells and cardiovascular system in general [44,45]. Receptors of NGF and VEGF activate a complex and integrated network of common signaling pathways in neurons and endothelial cells. Dysregulation of NGF signaling leads to increased TrkA signaling, which promotes proliferation, mitogenesis, invasion, metastasis, and angiogenesis by activating MAPK, PI3K/Akt, and PLCγ pathways [46,47], in non-neuronal carcinomas such as breast cancer.

SEMA3A in the “drug response (baseline) N38” module is involved in angiogenesis. SEMA3A has stimulatory and inhibitory effects on angiogenesis, depending on the environment [48,49]. Semaphorins signal through two major receptor families, plexins and neuropilins (NRP), SEMA3A uniquely binds to NRP1. Both NRP1 and SEMA3A are involved in the immune system. NRP1 is an immunoregulatory receptor that has received renewed attention as it reinforces the function of the intratumoral regulatory T cells (T_regs_) function for inflammation in the TME [50]. T_regs_ are associated with angiogenesis. SEMA3A contributes to immune suppression in tumors by impairing the function of the CD8^+^ T cells [51].

The network module scores of the “drug response (baseline) N8” module (S100A family), “drug response (baseline) N19” module (NGF pathway), and “drug response (baseline) N38” module (SEMA family) showed a moderate anti-correlation with the antitumor activity, indicating that a lower module score is associated with higher antitumor activity (i.e., “sensitive” group). The activation of angiogenesis by factors other than those directly targeted by lenvatinib constitutes an intrinsic resistance mechanism. This could explain the higher scores of the mouse models in these network modules resulting in no or weaker antitumor effect following lenvatinib treatment. SEMA3A and S100A4 were targeted in previous studies with animal models [48]. Anti-SEMA3A antibody demonstrated tumor inhibitory effects by downregulating TAM recruitment in a glioblastoma PDX model [52]. Anti-S100A4 monoclonal antibody blocks the attraction of T cells to the fibroblast monolayer in the lungs of a mouse model, thereby suppressing metastasis [53]. An anti-S100A4-neutralizing antibody reduced tumor angiogenesis and growth in melanoma and pancreatic mouse xenograft models [54].

Other mechanisms associated with lenvatinib include the interleukin (IL) pathway. IL-18 promotes angiogenesis through the activation of NF-κB, thereby triggering the secretion of VEGF from cancer cells, inducing their proliferation and invasion with preventing apoptosis. IL-18 may also induce PD-1-dependent immunosuppression of natural killer cells [55]. It was a biomarker associated with progression-free survival in a phase 2 trial of lenvatinib + everolimus in patients with metastatic renal cell carcinoma [56]. The functions enriched in the network modules crucial for predicting lenvatinib response were related to angiogenesis and involved in crosstalk with the immune system. This is consistent with the findings of the study by Kato et al. [31], which demonstrated that the antitumor effect of lenvatinib is linked to the modulation of the immune system in the TME, as well as angiogenesis inhibition.

The Wnt signaling pathway regulates various cellular processes including determination of the fate of the cell, proliferation, differentiation, and apoptosis. Wnt signaling pathway is involved in the malignancy of cancers such as immune escape; thus, it is targeted as a potential therapeutic candidate [57,58]. LGR4, LGR5, and LGR6 in the “drug response (baseline) N40” module are receptors to interact with RSPO proteins also found in the same network module to enhance Wnt/β-catenin signaling. Independently, a recent pharmacogenomic study using patient-derived liver cancer organoids identified aberrant Wnt/β-catenin signaling as a contributor to lenvatinib resistance [59], providing orthogonal functional evidence that this pathway is implicated in lenvatinib response, consistent with the enrichment of LGR4/5/6 and RSPO proteins in our network module.

### 3.3. Differential Contributions of Cancer-Cell and Host-Cell Modules

The cancer genes were primarily linked to “drug response” network modules when predicting drug responses. These modules encompass the signaling pathways, such as angiogenesis and immune systems, that are directly associated with the mechanism of lenvatinib. In contrast, the host genes were more closely associated with the “cancer progression” network modules, such as energy metabolism and regulation of cell motility, which are critical pathways for the proliferation phase. Notably, many cancer progression-related network modules derived from the prediction of host genes attained a high rank in the prediction of TCGA LIHC patient data. Thus, these features may reflect the characteristics of the cancer and host, rather than just being attributable to species differences. While the results from the host genes shared some characteristics with those of TCGA LIHC patient samples, it is important to note that the compromised adaptive immune system in the HCC PDX models hinders the evaluation of lenvatinib’s antitumor effect as an immune modulator.

### 3.4. Implications of the Limited Contribution of Drug Treatment Network Modules

For both cancer and host gene predictions in the HCC PDX models, adding “drug treatment” network modules to the best-performing feature sets did not enhance and often reduced predictive performance. Within a framework in which all ML features were calculated using pre-treatment RNA-seq profiles, the reduced contribution of “drug treatment” network modules, defined based on treatment-induced expression changes, suggests that baseline transcriptional states may be more informative for stratifying lenvatinib response than treatment-induced transcriptional changes. Rather, these modules primarily reflect downstream gene expression patterns associated with pathway activation following drug treatment, whereas network modules related to drug response or cancer progression more directly capture transcriptional states relevant to treatment outcome prediction.

### 3.5. Limitations and Future Directions

The conclusions of this study should be interpreted in light of several key limitations.

First, the ML model-based framework relies on relatively small mouse model datasets: syngeneic mouse models (36 data points) and HCC PDX mouse models (66 data points for cancer genes; 15 data points for host genes). These sample sizes inherently limit statistical power. Class imbalance between lenvatinib “sensitive” and “non-sensitive” groups and the high dimensionality of module-level features also introduce a risk of overfitting. To mitigate these issues, we adopted several strategies in the modeling pipeline. Hyperparameters were narrowed using the HCC PDX dataset strictly as a development test set, without allowing HCC PDX data to influence model training, thereby reducing the effective search space. We performed 100 iterations of three-fold cross-validation on the syngeneic mouse data and used stratified splits to preserve the sensitive/non-sensitive ratio in each iteration. These procedures increase the robustness of performance estimates but cannot fully eliminate the possibility of overfitting. In this context, our framework should be viewed as exploratory and hypothesis generating rather than as a fully powered predictive model. Importantly, sensitivity analyses indicated that the key network-level signals identified in this study were robust to modest uncertainty in phenotype classification, supporting the internal stability of the framework despite these data limitations.

Second, the syngeneic training panel includes multiple non-HCC tumor lineages. Because sufficient lenvatinib-treated, pre-treatment RNA-seq datasets are not available for HCC-only mouse cohorts, the present models were trained on a multi-tissue syngeneic panel. Aggregating gene expression into diffusion-derived network-module scores partially reduces tissue-specific noise by emphasizing conserved biological processes instead of individual genes. However, the current data do not allow us to determine how much of the learned signal is tissue specific versus tissue agnostic. Accordingly, some of the identified network modules may reflect partially tissue-agnostic drug response or stress-related programs rather than strictly HCC-specific mechanisms. Future work will benefit from expanded PDX resources across multiple tissues, each with lenvatinib treatment and matched transcriptomes, which would enable explicit evaluation of tissue-specific versus generalizable pharmacologic mechanisms.

Third, datasets suitable for rigorous human validation remain limited. To our knowledge, available LIHC clinical datasets that include paired pre-treatment RNA-seq and lenvatinib outcomes are limited in size and therefore insufficient for meaningful ML-based validation. Moreover, the TCGA LIHC dataset does not include lenvatinib-treated patients or treatment outcomes and therefore cannot serve as a true external validation cohort. Accordingly, TCGA data were not used for clinical validation but rather as an independent human transcriptomic context with pseudo-labels to assess whether network modules identified in the HCC PDX setting correspond to dominant patterns of transcriptional variation in human tumors. Definitive clinical validation will require future LIHC cohorts with paired pre-treatment RNA-seq and lenvatinib response data. While several recent studies have examined lenvatinib response in HCC using diverse experimental systems, including neoadjuvant surgical specimens, cell line-based resistance models, targeted immune gene panels, and patient-derived organoid models, these studies provide valuable biological insights but differ from our framework in sample type, scale, or response annotation [59,60,61,62,63].

Even within mouse systems, datasets combining transcriptomic profiles with lenvatinib antitumor activity are scarce. The syngeneic mouse panel [32] is one of the few publicly available resources with matched RNA-seq and detailed outcome measurements. Consequently, the HCC PDX panel was effectively the only independent dataset with both transcriptomic data and lenvatinib responses and therefore served as the sole out-of-training comparator. We note that the compromised adaptive immune system in HCC PDX models introduces an additional constraint, particularly for evaluation of lenvatinib’s antitumor effect as an immune modulator. As with any computational study that relies on experimental data from animal models, the biological validity of the gene signature identified here is inherently contingent on the quality and reproducibility of the underlying in vivo experiments.

Because HCC PDX models lack adaptive human immunity, we do not attempt to interpret human immune mechanisms directly from these systems. Instead, human transcriptomic data were used as a contextual reference to identify network-level transcriptional signals derived from PDX analyses that are also observable in human tumors. This cross-context comparison was intended to reduce the influence of mouse-specific effects, thereby prioritizing conserved transcriptional network features rather than serving as clinical validation.

Finally, despite these limitations, a key motivation of this study was to test whether any transcriptional features predictive in mouse models would exhibit coherent behavior in human liver tumors. Several network modules that repeatedly contributed to predictions in both syngeneic and HCC PDX models also emerged as important in TCGA LIHC transcriptomes. Many of these shared modules correspond to biological functions linked to lenvatinib’s mechanism, including angiogenesis-related and immune-associated pathways. This convergence suggests that the identified modules are not purely mouse-specific artifacts but may reflect conserved drug-response biology. Moreover, the value of these multi-cancer-type syngeneic models, generated under well-controlled experimental conditions, is underscored by their ability to help dissect TME–drug interactions in a systematic manner. A comprehensive collection of syngeneic mouse tumor expression profiles under various immunotherapy treatments has been developed recently [64]. As additional mouse model and clinical patient datasets become available, the framework proposed here can be extended and refined, for example by building tissue-specific models for different cancer types and by systematically comparing models across multiple targeted agents and immunotherapies. Such efforts may help move toward more robust biomarker development and precision use of lenvatinib and related therapies.

## 4. Methods

### 4.1. Syngeneic Mouse Tumor Models: Classification Based on the Antitumor Activity of Lenvatinib

The change in tumor volume following treatment with lenvatinib was used to determine the antitumor activity of lenvatinib in a panel of syngeneic mouse tumor models, as previously reported [32].

For each mouse, the tumor volume on day *N* was divided by the tumor volume on day 1 to obtain the relative tumor volume (*RTV* = *TV_N_*/*TV*_1_). *RTV_TN_* and *RTV_CN_* denote the mean *RTV*s of the treated (T) and control (C) groups, respectively, on day *N* after treatment initiation.

Δ*T*/*C* (%) was calculated following the definition used in [32], which evaluates antitumor activity by comparing the relative changes in tumor growth between treated and control groups. Consistent with that definition, the Δ*T*/*C* (%) was computed as:

$$∆\frac{T}{C}=\;\frac{\left({RTV}_{TN}-\;1\right)\times 100}{{RTV}_{CN}-\;1}.$$

When *RTV_TN_* fell below 1 (indicating shrinkage below baseline tumor size), Δ*T*/*C* was calculated as:

$$∆\frac{T}{C}=\left({RTV}_{TN}-1\right)\times 100.$$

Tumor volumes in the syngeneic mouse studies were recorded throughout the standard observation period of the original efficacy experiment. The total number of days differed among syngeneic models and generally ranged within a 3–4-week observation window, depending on tumor growth and humane endpoints. Tumor volume data from mice that died on that day were excluded from *RTV* and Δ*T*/*C* calculations. The syngeneic models with a minimum Δ*T*/*C* of <10% were classified as lenvatinib “sensitive.” The remaining models were classified as “non-sensitive.”

### 4.2. Syngeneic Mouse Tumor Models: Transcriptomic Data

The RNA-seq dataset of the syngeneic mouse tumor models analyzed in this study was obtained in a previous study [32] and is publicly available in the Gene Expression Omnibus (GEO; RRID:SCR_005012) under accession number GSE281579.

In total, 12 syngeneic mouse tumor models were included. For each model, tumors were sampled under three experimental conditions: tumor tissues resected when the tumor volume reached approximately 100 mm^3^ (“baseline”), tumors collected after 7 days without any treatment (“non-treatment”), and tumors collected after 7 days of lenvatinib administration (“treated”). Each condition comprised three biological replicates, yielding 108 RNA-seq samples in total (12 models × 3 conditions × 3 replicates).

### 4.3. HCC PDX Models: Antitumor Activity of Lenvatinib

Tumor tissues from 22 human HCC cases were engrafted subcutaneously into nude mice by Crown Bioscience (Beijing, China) to establish HCC PDX models, following standardized protocols. Nine-week-old female BALB/c nude mice were purchased from Beijing Anikeeper Biotech Co., Ltd. (Beijing, China) for in vivo studies. Detailed descriptions of the HCC PDX models, including model characteristics, are publicly available in the Crown Bioscience PDX model database. The antitumor effects of lenvatinib were evaluated at Crown Bioscience after tumors reached 100–200 mm^3^, in accordance with AAALAC International standards.

In the original efficacy experiment, a total of 660 animals underwent experimental procedures across five treatment groups for the 22 PDX models. For the present analysis, we used only the two groups relevant to evaluating lenvatinib monotherapy: the non-treatment (control) group and the lenvatinib group. Thus, the dataset analyzed in this study consisted of 132 animals (22 models × 2 groups × 3 mice).

Randomization was performed using the “Matched distribution” randomization method via a multi-task method (StudyDirectorTM software, version 3.1.399.19) on day 1. No formal sample size calculation was conducted, as the sample size was determined to enable the evaluation of antitumor efficacy across multiple independent HCC PDX models.

Lenvatinib (10 mg/kg) was administered orally once daily (QD). The tumor volumes were measured twice weekly in two dimensions using a caliper and expressed in mm^3^ using the following formula:

*V* = (*L* × *W* × *W*)/2

where *V*, *L*, and *W* denote the tumor volume, tumor length (longest tumor dimension), and tumor width (longest tumor dimension perpendicular to *L*), respectively. Relative body weight was calculated as the ratio of body weight measured each day to that recorded on day 1. Mice were given a dosing break until their weight returned to within 10% of baseline.

The administration of lenvatinib was continued for three weeks, and tumor samples were collected on the final day. Mice were euthanized under the following conditions: (i) the tumor volume of any individual mouse exceeded 3000 mm^3^; (ii) the mean tumor volume of a group exceeded 2000 mm^3^; or (iii) body weight loss reached ≥20% from baseline. Tumors were then harvested. The tumor samples were frozen immediately after removal using liquid nitrogen and stored at −80 °C until the RNA-seq analysis.

For the HCC PDX models, tumor volumes were recorded through day 28. The Δ*T*/*C* values were computed using the same procedure as applied to the syngeneic mouse models, and the models were classified as lenvatinib “sensitive” or “non-sensitive” using the same threshold (minimum Δ*T*/*C* < 10%).

### 4.4. HCC PDX Models: RNA-Seq

Sequencing libraries were generated using RNAs from the tumor tissues with an Illumina TruSeq Stranded Total RNA with a Ribo-Zero Globin kit (Illumina, San Diego, CA, USA) in accordance with the manufacturer’s instructions. Paired-end sequencing (2 × 150 bp) was conducted using Illumina NovaSeq in accordance with the manufacturer’s instructions. Trimmomatic (v0.36) was used to process the sequencing data to remove all Illumina adaptor sequences and bases with low-quality scores. The filtered reads were mapped to human genome (GRCh38) and mouse genome (mm10) using STAR (v2.5.2b). RSEM (v1.2.31) was used to estimate gene expression levels.

RNA-seq was performed for 22 HCC PDX models in two groups (non-treatment and lenvatinib-treated) with three biological replicates per group, resulting in 132 tumor RNA-seq samples (22 models × 2 groups × 3 replicates). However, since only the RNA-seq data from the non-treatment group was used in this study, a total of 66 samples were listed in Table S2. For each sample, separate expression profiles were obtained for human (cancer) genes and mouse (host) genes. Quality assessment of the RNA-seq data was performed, and only a subset of the data mapped to the mouse genome was used in the present study (see Section S1 in Supplementary Material for further details).

### 4.5. TCGA Liver Hepatocellular Carcinoma Cohort: Transcriptomic Data

The Cancer Genome Atlas (TCGA) [41,42] is a database of genomic data on various types of cancer that includes RNA-seq datasets derived from clinical samples. A set of RNA-seq expression profiles (*n* = 371) of tumor samples from patients with liver hepatocellular carcinoma (LIHC) was downloaded from the Broad GDAC Firehose. The corresponding TCGA sample identifiers are provided in Table S6. The read counts of genes and TPM values produced using the RSEM program were included in the downloaded RNA-seq data.

### 4.6. Identification of Non-Canonical Network Modules by HotNet2

Gene subsets that changed with cancer progression or in response to treatment and potential factors that indicate differences in drug responsiveness of syngeneic mouse tumor models were extracted using a network-diffusion-based algorithm, HotNet2 [33]. HotNet2 was originally developed for identifying subnetworks enriched for somatic mutations; however, the algorithm implements a general heat-diffusion process over a vertex-weighted interaction network and therefore accepts any continuous gene-level score as input. In this study, log fold-change values derived from transcriptomic comparisons were used as node weights.

PPI data were obtained from HiNT [65]. FGF–FGFR interactions, which involve direct targets of lenvatinib and were incompletely represented in HiNT, were supplemented using data from [66] to construct the reference network. Three categories of the “non-canonical” network modules were defined as follows. Here, the term “non-canonical” refers to network modules identified in a data-driven manner by network diffusion, rather than predefined pathways or curated gene sets.

(a) Cancer progression

To determine the changes in expression levels that occur during cancer progression, we used expression data from two time points: “baseline” (early-stage tumors) and “non-treatment” (later-stage tumors, prior to drug treatment). For each mouse model, the average TPM values were calculated from biological triplicates at each time point. Then, the log-transformed fold-change of “non-treatment” over “baseline” TPMs was calculated. These values were served as input to define network modules of “cancer progression.”

(b) Drug treatment

To determine the changes in expression levels that occur owing to treatment with lenvatinib, we compared expression levels between the “non-treatment” and “treated” (with lenvatinib) conditions. For each mouse model, the average TPM values were calculated from biological triplicates at each condition. Then, the log-transformed fold-change of “treated” over “non-treatment” TPMs was calculated. These values were used to define network modules of “drug treatment.”

(c) Drug response

To determine the drug response potential, tumor models were classified as “sensitive” or “non-sensitive” based on the antitumor activity. For each group, the averaged TPM values of “baseline” or “non-treatment” were calculated. Then, the log-transformed fold-change of “sensitive” over “non-sensitive” was calculated for the “baseline” or “non-treatment” condition. These values were used to identify network modules of “drug response”, which are potential markers of drug responsiveness that can be observed prior to drug administration.

HotNet2 only accepts positive values; thus, the absolute fold-change values were used as the input values. To avoid artifacts arising from expression-derived node-weight distributions, candidate *δ* values were evaluated using HotNet2’s permutation-based significance testing, and those with *p* < 0.05 were considered statistically acceptable. When multiple *δ* values satisfied this criterion, we selected the δ that yielded subnetworks with a minimum size ≥ 5. The selected *δ* was then used to extract the final non-canonical subnetworks. When there was a complete overlap in the members of network modules, the overlapping modules were merged and treated as a single module.

### 4.7. Calculation of Non-Canonical Network Module Scores

Gene expression data from the syngeneic mouse models and the HCC PDX models were mapped to human gene symbols using a mouse–human ortholog table obtained from the Ensembl Genome Browser. HotNet2 was used to convert the RNA-seq TPM values from stationary heat scores into diffused heat scores. The TPM values were log_2_-transformed. HotNet2 does not accept values of ≤0; consequently, a negligibly small value was padded to the TPM values. The expression data of “baseline” were used in the syngeneic mouse models (36 data points), whereas the “non-treatment” from the HCC PDX models (66 data points for cancer genes; 15 data points for host genes). All available TCGA LIHC expression data were used in the analysis. A gene-set overall expression approach was used to aggregate the diffused heat scores of its members to determine the score for each non-canonical network module [17].

### 4.8. Canonical Pathway and Transcription-Factor Activity Features

The ML models were implemented using the PROGENy pathways [38] and DoRothEA transcription factor (TF) regulons [39] as features. The member gene coefficient matrices for 14 pathways, comprising androgen, EGFR, estrogen, hypoxia, JAK-STAT, MAPK, NF-kB, p53, PI3K, TGF-b, TNF-a, Trail, VEGF, and WNT based on the pathway-perturbation experiments [38], were computed using PROGENy. The RNA-seq read counts of the member genes were input into the PROGENy software to calculate the pathway activities. A comprehensive set of TF-target interactions for 115 TFs was collected from the literature and databases, and TF regulon activities were estimated using DoRothEA by inputting the RNA-seq TPM data of the target genes of TFs [39].

### 4.9. Machine Learning Model Construction and Evaluation

ML models were constructed separately on the three described non-canonical network modules (single views), pairwise combinations of two views, and all three views. Non-canonical network modules whose members matched completely or were covered by other network modules were excluded from the input features for each view. The number of non-canonical network modules used for combinations of two views were as follows: “cancer progression” + “drug treatment,” 144; “cancer progression” + “drug response,” 142; and “drug treatment” + “drug response,” 102. The 184 non-canonical network modules used for all combinations of the three views were listed. The full hyperparameter search space, cross-validation procedure, and the selected optimal algorithms and parameters for each feature view are described in Supplementary Material (Section S2). Statistical comparison of classifier prediction performance across feature views (ANOVA + Tukey’s HSD) is provided in Supplementary Material (Section S3).

### 4.10. SHAP-Based Interpretation and TCGA-Based Qualitative Assessment

To interpret how individual non-canonical network modules contributed to model predictions, we computed Shapley values using the SHapley Additive exPlanations (SHAP) framework. For each ML model, module importance was quantified based on its Shapley value profile, and key contributors were identified by averaging their rankings across predictive models. A full description of the SHAP-based ranking procedure is provided in Supplementary Material (Section S4).

The information on drug responsiveness was not available in the TCGA LIHC dataset. Therefore, the ML models were initially applied to all samples, and the predicted outcomes were treated as proxy labels for qualitative stratification, rather than true measures of antitumor activity. The samples with more robust and consistent predictions were obtained for the “sensitive” and “non-sensitive” groups, respectively. SHAP analysis was performed on the selected TCGA LIHC samples to identify important non-canonical network modules contributing to their classification, which were considered to reflect transcriptional modules highlighted by mouse-trained models when applied to human TCGA LIHC transcriptomes, without implying therapeutic efficacy.

The 300 ML models produced from 100 iterations of three-fold cross-validation on the syngeneic mouse data on three views (“drug response,” “drug response plus cancer progression,” and “all”) with all available LIHC samples were used to make the predictions. The parameters used for the ML models were identical to those used for predicting cancer genes in tumors from the HCC PDX models.

The majority of LIHC samples were classified as “non-sensitive.” However, a notable proportion were consistently classified as “sensitive” by almost all 300 models. SHAP analysis was performed using the top 30 samples predicted to be lenvatinib-sensitive by the most predictive models and 30 samples that were randomly selected from the predicted non-sensitive samples by all 300 models. The important non-canonical network modules of these 60 selected samples across the 300 predictive models were analyzed.

### 4.11. Functional Term Enrichment Analysis by DAVID

The important non-canonical network modules for the antitumor effects of lenvatinib were analyzed using DAVID webtools (accessed in 2023–2024) to determine their effects on biological function [67,68,69]. DAVID recruits the annotated terms for genes from various databases. Eight additional categories (UP_ACTIVITY_REGULATION, UP_CATALYTIC_ACTIVITY, UP_FUNCTION, UP_INDUCTION, UP_PATHWAY, UP_PTM, REACTOME_PATHWAY, and WIKIPATHWAYS) were selected for functional term enrichment analysis in addition to the default inclusion of DAVID. The false discovery rate was controlled to <0.1 to identify significantly enriched functional terms. The redundant and significantly enriched terms of an important non-canonical network module were merged according to the clustering results of DAVID [69].

## Figures and Tables

**Table 1:** Important non-canonical network modules for predicting sensitivity to lenvatinib.

Network Module Name	PDX-TCGA Overlap	
	PDX Cell Type	Class
Drug response (baseline) N8	Cancer	I
Drug response (baseline) N19	Cancer	I
Drug response (baseline) N34	Cancer	I
Drug response (baseline) N40	Cancer	I
Cancer progression (4T1) N17	Cancer	I
Drug response (baseline) N2	Cancer	II
Drug response (baseline) N28	Cancer	II
Drug response (baseline) N38	Cancer	II
Drug response (baseline) N42	Cancer	II
Drug response (non-treatment) N1	Cancer	II
Cancer progression (4T1) N18	Host	II
Cancer progression (4T1) N32	Host	II
Cancer progression (4T1) N50	Host	II
Cancer progression (BNL) N3	Host	II
Cancer progression (EMT6) N5	Host	II

## Data Availability

The sequence data and tumor volume data generated in this study are not publicly available but are available upon reasonable request from the corresponding authors. Other data generated in this study are available in the article and the Supplementary Data Files. The code for calculating network module score is available at https://bitbucket.org/sbijapan/network-module-score-calculation_release/src/main/ (accessed on 21 December 2025). The code for machine learning model building and evaluation of key network modules with SHAP analysis is available at https://bitbucket.org/sbijapan/ml-model-training-and-evaluation_release/src/main/ (accessed on 21 December 2025).

## Supplementary Materials

The following supporting information can be downloaded at: https://www.mdpi.com/article/10.3390/cancers18071067/s1, Figure S1: Effect on tumor volume of the HCC PDX models with lenvatinib treatment. The color in the bar chart indicates the classification as sensitive (red) or non-sensitive (blue) based on the minimum Δ*T*/*C* in the time series with 10% as the threshold (dashed line).; Figure S2: Comparison of the performance of the machine learning models trained using features defined based on prior knowledge. A: Training performance for the cancer genes in the tumors from the HCC PDX models. B: Prediction performance for the cancer genes in the tumors from the HCC PDX models. C: Training performance for the host genes in the tumors from the HCC PDX models. D: Prediction performance for the host genes in the tumors from the HCC PDX models. A and C demonstrate the training performance over 300 iterations. B and D show the predictive performances of the 300 ML models. In these figures, the left panels illustrate the results of the AUC score, whereas the right panels show the results of the kappa score.; Figure S3: Saturation assessment of gene detection for the genes in the tumors from HCC PDX models. The green curves show the accumulation of newly detected Ensembl gene IDs with increasing sequencing depth for the human genes, while the red curves show the same for the mouse genes. The “k” on the y-axis represents thousand (kilo). The x-axis is in million of reads.; Figure S4: The optimal hyperparameter and algorithms for each input feature. A: Hyperparameter optimization based on kappa scores. The results of hyperparameter optimization for “drug response” single view and a pairwise combination “cancer progression plus drug response” were shown as examples. B: Algorithms and parameters selected for each view. The best performed ML classifier and their optimal hyperparameters for each view were identified for the prediction of the cancer genes and the host genes of the tumor in HCC PDX models, respectively.; Figure S5: Scheme of SHAP score-based identification of key non-canonical network modules for lenvatinib response.; Table S1: List of non-canonical network modules. Each sheet corresponds to the input type of non-canonical network modules (“cancer progression,” “drug treatment,” and “drug response”). Key genes related to angiogenesis, genes related to T-cell inflammation, and T-cell or tumor associated macrophages phenotype markers are marked.; Table S2: RNA sequencing data (TPM values) of human and mouse genes in tumors from the HCC PDX models.; Table S3: Number of predictive ML models with kappa scores of >0.5 for cancer genes or host genes in tumors from the HCC PDX models.; Table S4: Important non-canonical network modules for predicting sensitivity to lenvatinib (average ranking < 15). Related to Table 1; Table S5: Pathway enriched analysis of the network modules in Table 1. List of member genes of non-canonical network modules and enriched terms based on the DAVID analysis. No enriched terms were identified for “drug response (baseline) N34” and “drug response (baseline) N42.”; Table S6: TCGA sample identifiers for the LIHC RNA-seq dataset used in this study. List of TCGA barcode IDs corresponding to the 371 liver hepatocellular carcinoma (LIHC) tumor samples obtained from the Broad GDAC Firehose and analyzed in this study.; Table S7: The 15 HCC PDX model samples of which host genes in the tumor were qualified.; Table S8: ANOVA/Tukey adjusted *p*-values for pairwise comparisons among seven non-canonical feature-views (cancer-gene classifiers).; Table S9: ANOVA/Tukey adjusted *p*-values for pairwise comparisons among seven non-canonical feature-views (host-gene classifiers).; Table S10: Average ranking stability of “drug response” network modules across Δ*T*/*C* thresholds. Average ranking of “drug response” network modules in Table 1 was evaluated under Δ*T*/*C* thresholds of 10% and 12%. “Enriched Function” indicates representative functional terms derived from functional term enrichment analysis (see Table S5 for details).; Table S11: Reproducibility of “drug response” network modules across label-perturbation analyses. Modules listed in Table 1 are shown with their enriched functions and the frequency of appearance within the top 20 ranked features across (i) valid label-perturbation runs (kappa score > 0.5; 26 runs) and (ii) perturbation runs after excluding those without effective label changes (21 runs). “Enriched Function” indicates representative functional terms derived from functional term enrichment analysis (see Table S5 for details).; Table S12: Hypergeometric test results for overlap of highly ranked network modules (average rank < 15) between HCC PDX and TCGA LIHC across comparable ML input views, including raw *p*-values and FDR-adjusted *q*-values. Supplementary Material File: Detailed supplementary methods and analyses related to this study [70,71,72].
