# Supplementary material for: Leveraging Network-Based Transcriptome Analysis from Mouse Tumor Models and Explainable Artificial Intelligence to Advance the Understanding of the Antitumor Activity of Lenvatinib
Source: Cancers (Basel). 2026 Mar 25;18(7):1067. doi: 10.3390/cancers18071067 (PMC13072315; doi:10.3390/cancers18071067)
Supplement: Supplementary file 1 [file cancers-18-01067-s001.zip › cancers-4086555-supplementary.pdf]

Supplementary Figures

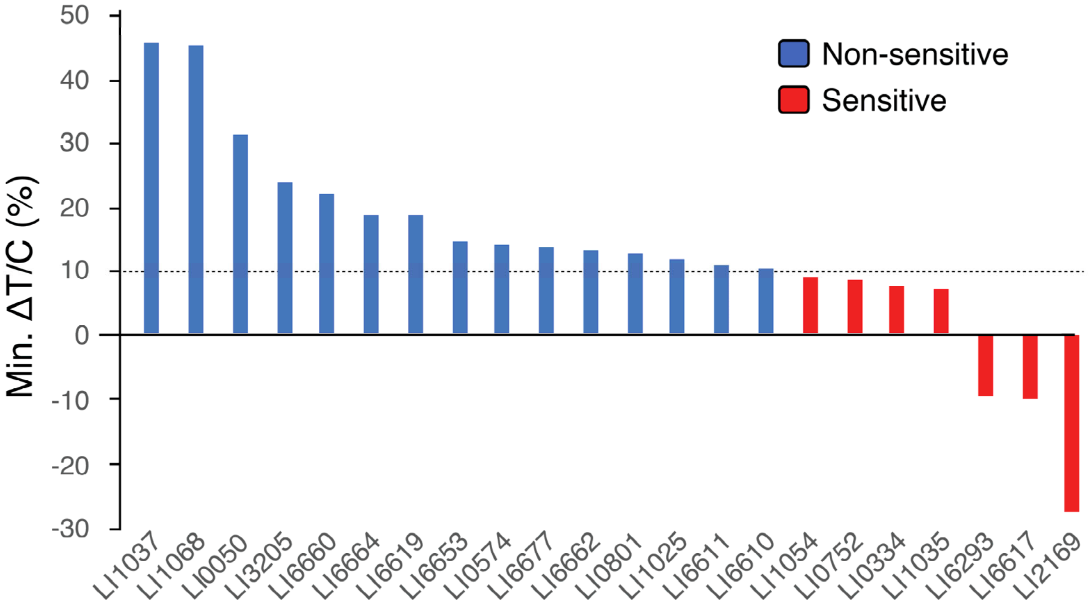

Figure S1. Effect on tumor volume of the HCC PDX models with lenvatinib treatment. The color in the bar chart indicates the classification as sensitive (red) or non-sensitive (blue) based on the minimum ΔT/C in the time series with 10% as the threshold (dashed line).

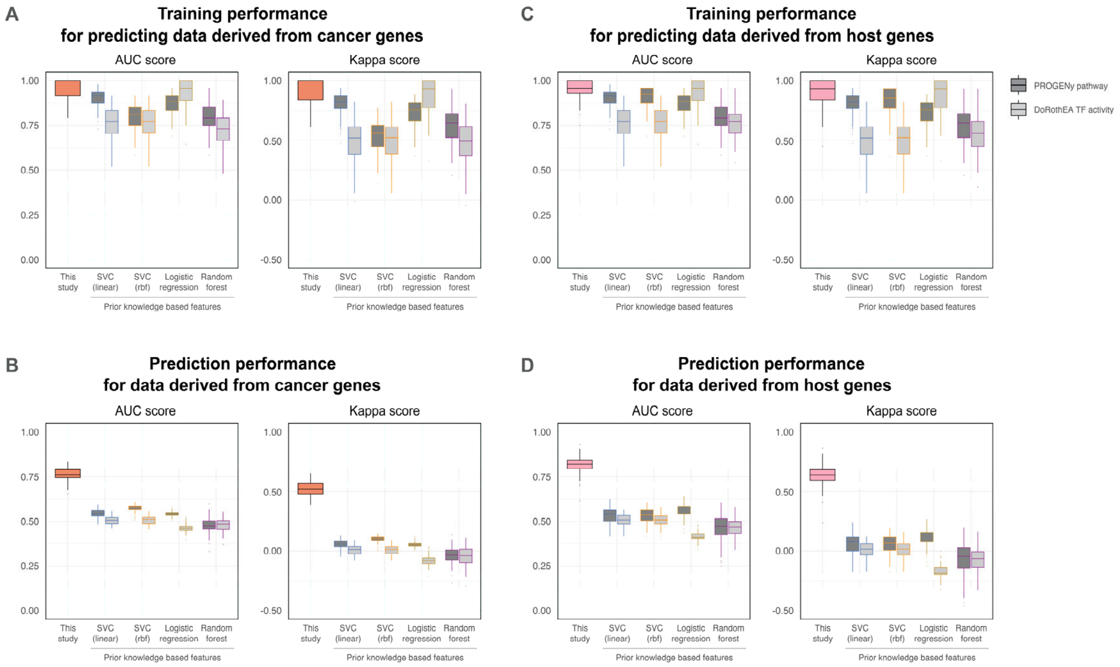

Figure S2. Comparison of the performance of the machine learning models trained using features defined based on prior knowledge. A: Training performance for the cancer genes in the tumors from the HCC PDX models. B: Prediction performance for the cancer genes in the tumors from the HCC PDX models. C: Training performance for the host genes in the tumors from the HCC PDX models. D: Prediction performance for the host genes in the tumors from the HCC PDX models. A and C demonstrate the training performance over 300

iterations. B and D show the predictive performances of the 300 ML models. In these figures, the left panels illustrate the results of the AUC score, whereas the right panels show the results of the kappa score.

Supplementary Tables

Table S1. (attached). List of non-canonical network modules. Each sheet corresponds to the input type of non-canonical network modules (“cancer progression,” “drug treatment,” and “drug response”). Key genes related to angiogenesis, genes related to T-cell inflammation, and T-cell or tumor associated macrophages phenotype markers are marked.

Table S2. (attached). RNA sequencing data (TPM values) of human and mouse genes in tumors from the HCC PDX models.

Table S3. Number of predictive ML models with kappa scores of >0.5 for cancer genes or host genes in tumors from the HCC PDX models.

	DR	CP	DT	DR+CP	DR+DT	CP+DT	All (DR+CP+DT)
Cancer genes in the HCC PDX models	76	0	0	191	23	0	179
Host genes in the HCC PDX models	5	223	0	222	31	180	178

DR: drug response, CP: cancer progression, DT: drug treatment.

Table S4. (attached). Important non-canonical network modules for predicting sensitivity to lenvatinib (average ranking < 15). Related to Table 1.

Table S5. (attached). Pathway enriched analysis of the network modules in Table 1. List of member genes of non-canonical network modules and enriched terms based on the DAVID analysis. No enriched terms were identified for “drug response (baseline) N34” and “drug response (baseline) N42.”.

Table S6. (attached). TCGA sample identifiers for the LIHC RNA-seq dataset used in this study. List of TCGA barcode IDs corresponding to the 371 liver hepatocellular carcinoma (LIHC) tumor samples obtained from the Broad GDAC Firehose and analyzed in this study.
