# Supplementary material for: Leveraging Network-Based Transcriptome Analysis from Mouse Tumor Models and Explainable Artificial Intelligence to Advance the Understanding of the Antitumor Activity of Lenvatinib
Source: Cancers (Basel). 2026 Mar 25;18(7):1067. doi: 10.3390/cancers18071067 (PMC13072315; doi:10.3390/cancers18071067)
Supplement: Supplementary file 1 [file cancers-18-01067-s001.zip › cancers-4086555-supplementary_revised.pdf]

S1. Quality assessment of RNA-seq data

Amount of mouse host cells in the resected samples is lower compared to the human cancer cells in the tumors from HCC PDX models. Therefore, the absolute number of reads derived from the mouse host cells are lower than those of the human cancer cells. Given that there may be sensitivity issues with the results mapped to the mouse genome, the saturation curve analysis [70] was performed to access the sequencing depth and the quality of the RNA-seq of the human genes and the mouse genes in the tumors from the HCC PDX models (Figure S3). Saturation curves with slower gradients indicate that increasing RNA-seq depth does not increase gene detection, suggesting that sequence depth is sufficient. The samples showed less than one gene per 1000 reads in the last 50,000 reads of the saturation curve were defined as having sufficient sequence depth. The human genes from all 66 samples were measured at sufficient depth, while the mouse gene in 15 out of 66 samples were defined as having sufficient sequence depth (Table S7).

S2. Hyperparameter optimization and algorithm selection for machine learning model construction

The three-fold cross validation and grid search were performed on mouse syngeneic model data as a training set, and kappa scores were calculated to assess the prediction performance using HCC PDX data as a test set, with four supervised machine learning (ML) algorithms - including the support vector machines (SVMs) with linear kernel function and radial basis function (RBF), logistic regression (LR), and random forest (RF) (Figure S4A). The optimal hyperparameters and the algorithms of the best performing ML model were selected for the subsequent re-training (Figure S4B).

The syngeneic mouse model data were randomly split into three consecutive folds (three-fold cross-validation), preserving the sensitive/non-sensitive ratio. In each iteration, one-fold was used as a test set, while the remaining two formed a training set. The ML model was built on the training set. Kappa score and the area under receiver operating characteristic curve (AUC) score were calculated to assess the training and testing performance. The three-fold data split was repeated 100 times with random initial seeds. The ML models were further applied to predict cancer genes and host genes in the tumor from the HCC PDX models.

Construction and evaluation of the ML model were implemented using the Python Scikit-learn module [71].

S3. Statistical evaluation of ML model prediction performance

To systematically assess ML model prediction performance, we conducted statistical comparisons across both the non-canonical feature views and the canonical pathway- and transcription factor–based features. In addition to the AUC and kappa scores presented as Figures 3 and S2, we computed three additional performance metrics—accuracy, precision, and F1 score. For each metric, one-way ANOVA was performed followed by Tukey's HSD post-hoc tests to obtain adjusted *p*-values for the pairwise comparisons.

First, all pairwise comparisons among the seven non-canonical feature views (Figures 3; DR: drug response, CP: cancer progression, DT: drug treatment, DR+CP, DR+DT, CP+DT, and All: DR+CP+DT) were evaluated. The complete tables summarizing the adjusted *p*-values for each metric are provided in Tables S8 and S9. These results identify which view pairs exhibit statistically significant performance differences and support the predictive performance trends described in the main text.

Second, to compare the non-canonical network modules with canonical feature sets (Figure S2), we conducted the same statistical evaluation for (i) non-canonical modules versus PROGENy pathway activity scores and (ii) non-canonical modules versus DoRothEA transcription factor activity scores. In both comparisons, the non-canonical modules demonstrated significantly higher predictive performance (adjusted $p < 0.001$), consistent with the differences visually observed in Figure S2. These findings further highlight the distinct advantage of the diffusion-derived non-canonical modules over canonical pathway- and TF-based activity scores.

S4. SHAP-based ranking of the important non-canonical network modules

The network modules contributing to the ML model prediction were ranked based on the SHapley Additive exPlanations (SHAP) analysis to interpret results and determine how each non-canonical network module influenced model outcomes (Figure S5). SHAP analysis was performed using Python SHAP Package [72]. Among the 300 ML models, those with kappa scores of >0.5 for the prediction of HCC PDX mouse model dataset were selected as "predictive" ML models. These models were used to identify non-canonical network module patterns associated with antitumor activity of lenvatinib. Modules with a Shapley value profile closer to the model predictions in Euclidean distance were considered more important. Thus, the importance of non-canonical network modules was ranked based on the Euclidean distance between their Shapley value profiles and the model predictions. Key modules contributing to lenvatinib's antitumor effects were prioritized by averaging their rankings across selected predictive ML models for HCC PDX models and TCGA LIHC samples.

S5. Sensitivity analysis of Δ*T*/*C* threshold selection

To evaluate the robustness of phenotype dichotomization based on Δ*T*/*C* values and to assess whether the identified key network modules (Table 1) are sensitive to the choice of classification threshold, we performed two complementary sensitivity analyses: (i) threshold-shift analysis and (ii) label-perturbation analysis.

For all sensitivity analyses described in this section, ML models were trained using "drug response" network module scores as input features and evaluated by predicting the drug response with cancer genes in the HCC PDX models.

To ensure consistency with the primary analysis, the training algorithm and hyperparameter settings were fixed across all sensitivity analyses. Specifically, models were trained using a linear SVM ($C = 0.5$), corresponding to the algorithm and hyperparameters selected in the main analysis (Figure S4B). Details of the algorithm selection and hyperparameter optimization procedure are provided in Section S2.

Threshold-shift analysis

First, we examined alternative Δ*T*/*C* thresholds (5%, 10%, 12%, and 15%) to assess their impact on class balance, model stability, and feature prioritization.

At a 5% threshold, the resulting classification was highly imbalanced (3 sensitive vs. 9 non-sensitive), leading to unstable model training and no ML models achieving sufficient agreement (kappa score >0.5) for reliable SHAP-based interpretation. Similarly, at a 15% threshold, although class balance was improved (6 sensitive vs. 6 non-sensitive), model performance was highly variable and no models met the kappa score >0.5 criterion, indicating reduced analytical stability under this classification.

At a 12% threshold, only 2 out of 300 trained ML models achieved kappa score >0.5, reflecting substantially reduced robustness compared with the 10% threshold. Nevertheless, comparison of average feature-ranking profiles revealed strong concordance between the 10% and 12% thresholds (Spearman's $r = 0.85$; Table S10). Importantly, the top-ranking network modules identified at the 10% threshold remained among the highest-ranked features at 12%, including "drug response (baseline) N8" (average rank 1.13 at 10% and 2.5 at 12%) and "drug response (baseline) N19" (average rank 2.26 at 10% and 1.0 at 12%).

Label-perturbation analysis

Second, to more directly assess robustness to minor uncertainty in phenotype assignment near the Δ*T*/*C* = 10% boundary, we performed a label-perturbation analysis. Borderline models were defined as those immediately above and below the Δ*T*/*C* = 10% threshold. For the syngeneic cohort (12 models), ±2 models (four total) were considered, whereas for the HCC PDX cohort (22 models), ±4 models (eight total) were considered. This choice introduced modest uncertainty localized near the threshold while preserving model stability.

In each perturbation run, class labels were randomly swapped for a subset of these borderline models with a probability of 30%, thereby introducing modest uncertainty in phenotype assignment while avoiding wholesale relabeling. For each perturbed label configuration, the full ML training and SHAP interpretation pipeline was repeated. This procedure was performed across 50 independent perturbations.

Across these perturbations, SHAP-based interpretation was feasible in 26 runs with at least one ML model achieving kappa score >0.5, reflecting expected variability in model stability under perturbed labels. Aggregation of SHAP results across valid runs demonstrated consistent prioritization of key network modules: "drug response (baseline) N8" was identified within the top 20 ranked modules in 84.6% of runs, and "drug response (baseline) N19" in 100% of runs (Table S11).

Across the 50 perturbation runs, the number of ML models achieving kappa scores >0.5 varied between runs, reflecting expected sensitivity of model stability to label perturbation. In some runs, the random perturbation procedure resulted in no effective label changes relative to the original configuration, yielding model counts comparable to the unperturbed setting. To assess whether inclusion of such runs influenced the robustness conclusions, we repeated the calculation excluding perturbation runs without effective label changes (21 runs). The resulting frequencies of top-ranked modules were highly consistent with those obtained using all valid runs, and no qualitative differences were observed (Table S11).

Summary

Taken together, the threshold-shift and label-perturbation analyses support that the network-level signals identified in this study reflect stable transcriptional programs associated with lenvatinib response. Although the choice of $\Delta T/C$ threshold influences overall model stability, the key network modules highlighted in the main analysis are consistently prioritized across reasonable variations in phenotype definition, indicating that the reported findings are not artifacts of a specific threshold choice.

These sensitivity analyses were conducted within the primary analytical setting that underpins the main biological interpretation of the study (ML model with "drug response" network modules for cancer-genes prediction). Robustness under alternative module categories (cancer progression or drug treatment modules) and host-gene prediction was not evaluated and remains an important direction for future work.

S6. Statistical assessment of cross-dataset consistency between PDX and TCGA

To quantitatively evaluate whether the overlap of important network modules identified from HCC PDX models and TCGA LIHC exceeds random expectation, we performed hypergeometric tests based on SHAP-derived average feature rankings.

Network module importance was assessed using SHAP-based average rankings as described in S4. Modules with an average ranking < 15 were defined as highly prioritized. This threshold was applied consistently across all analyses.

Only ML input views that were directly comparable between the HCC PDX and TCGA LIHC analyses were included. Specifically, for cancer cell-derived expression data, the DR, DR+CP, and All views were evaluated, whereas for host cell-derived expression data, the DR+CP and All views were evaluated (Table S4).

For each comparable view, the statistical significance of overlap between highly ranked network modules in the HCC PDX and TCGA LIHC analyses was assessed using a hypergeometric test. The universe was defined as the full set of network modules evaluated in the corresponding ML input view. Successes were defined as

modules with an average ranking < 15 in each dataset. The overlap between the two sets of highly ranked modules was then tested against random expectation.

A total of five hypergeometric tests were performed across the comparable views (Table S12). Resulting p-values were corrected for multiple testing using the Benjamini-Hochberg false discovery rate (FDR) procedure. Modules that appeared in at least one comparison with FDR-adjusted $q < 0.05$ were considered to show statistically supported cross-dataset consistency.

Supplementary Figures

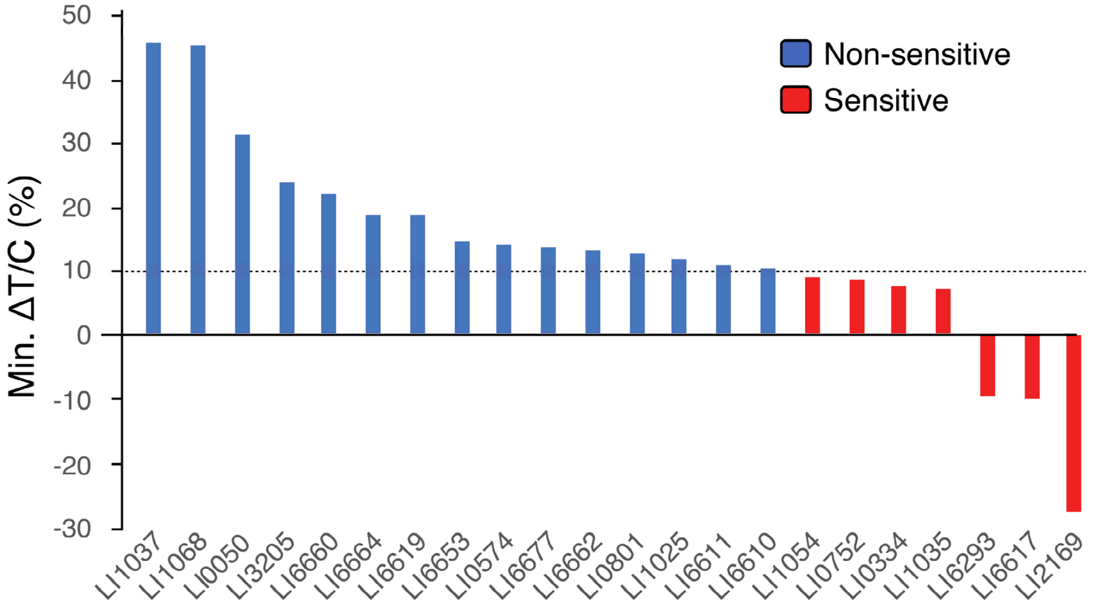

Figure S1. Effect on tumor volume of the HCC PDX models with lenvatinib treatment. The color in the bar chart indicates the classification as sensitive (red) or non-sensitive (blue) based on the minimum ΔT/C in the time series with 10% as the threshold (dashed line).

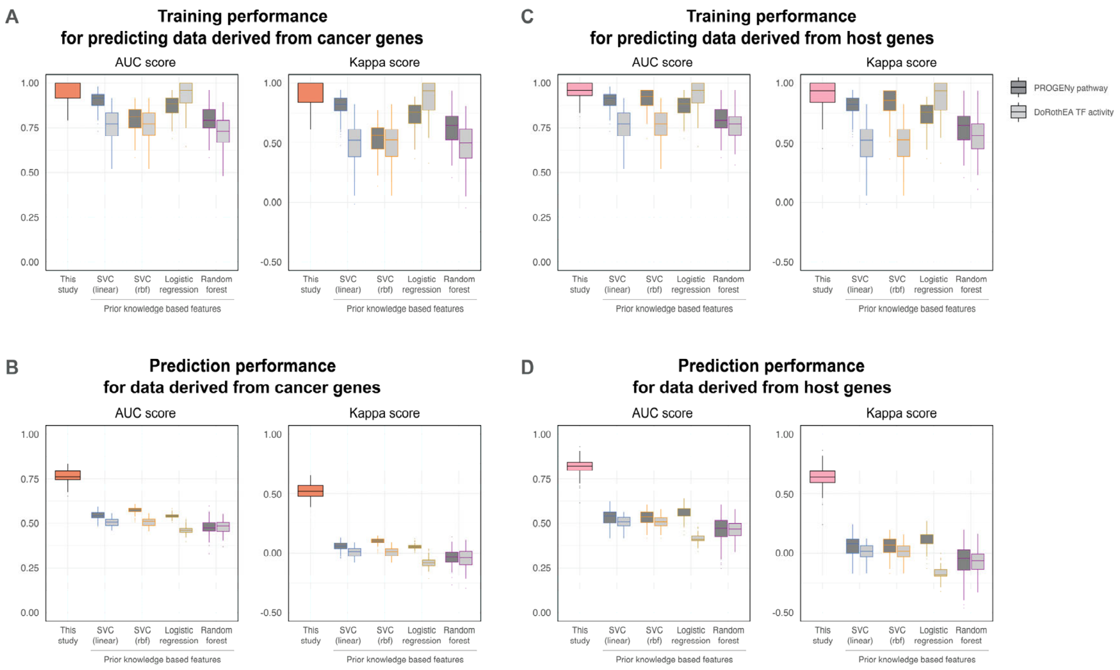

Figure S2. Comparison of the performance of the machine learning models trained using features defined based on prior knowledge. A: Training performance for the cancer genes in the tumors from the HCC PDX models. B: Prediction performance for the cancer genes in the tumors from the HCC PDX models. C: Training performance for the host genes in the tumors from the HCC PDX models. D: Prediction performance for the host genes in the tumors from the HCC PDX models. A and C demonstrate the training performance over 300 iterations. B and D show the predictive performances of the 300 ML models. In these figures, the left panels illustrate the results of the AUC score, whereas the right panels show the results of the kappa score.

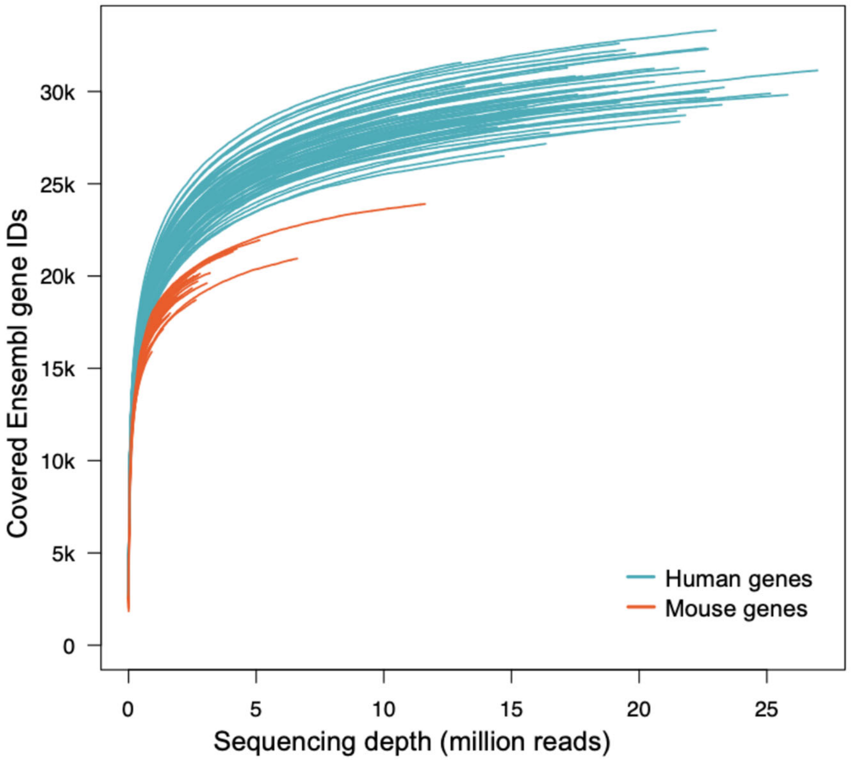

Figure S3. Saturation assessment of gene detection for the genes in the tumors from HCC PDX models. The green curves show the accumulation of newly detected Ensembl gene IDs with increasing sequencing depth for the human genes, while the red curves show the same for the mouse genes. The “k” on the y-axis represents thousand (kilo). The x-axis is in million of reads.

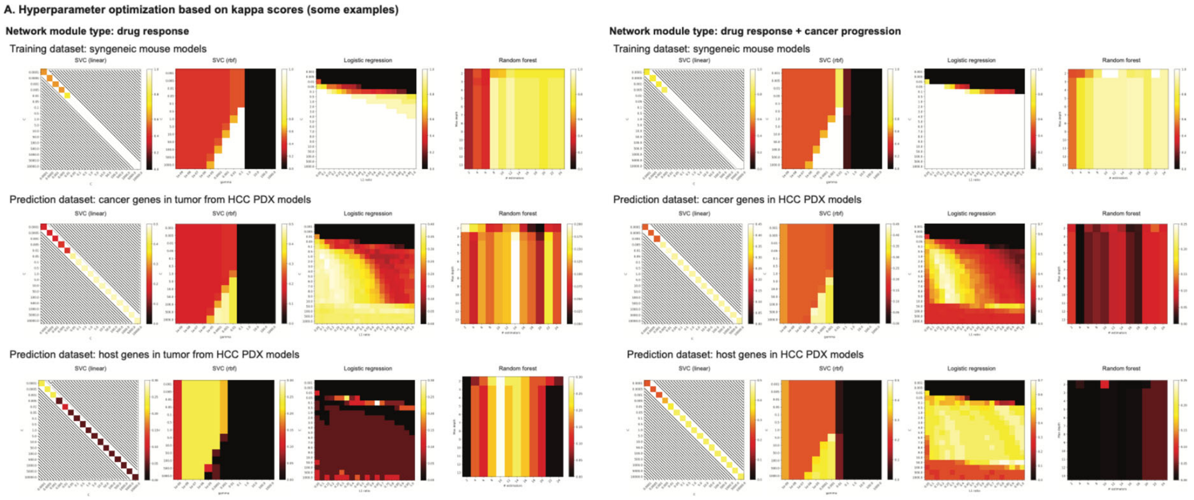

B. Algorithms and parameters selected for each input feature

Cancer genes in tumor from HCC PDX models

Network modules	Algorithm	Hyperparameters
Drug response	SVM (linear)	C=0.5
Cancer progression	Logistic regression	C=24, L1 ratio=0.6
Drug treatment	Logistic regression	C=0.3, L1 ratio=0.3
Drug response + cancer progression	Logistic regression	C=6.5, L1 ratio=0.225
Drug response + drug treatment	SVM (linear)	C=0.014
Cancer progression + drug treatment	Logistic regression	C=5, L1 ratio=0.5
All	Logistic regression	C=3, L1 ratio=0.15

Host genes in tumor from HCC PDX models

Network module type	Algorithm	Hyperparameters
Drug response	Logistic regression	C=0.105, L1 ratio=0.68
Cancer progression	Logistic regression	C=3, L1 ratio=0.85
Drug treatment	Logistic regression	C=25, L1 ratio=0.8
Drug response + cancer progression	Logistic regression	C=40, L1 ratio=0.7
Drug response + drug treatment	Logistic regression	C=45, L1 ratio=0.7
Cancer progression + drug treatment	Logistic regression	C=2, L1 ratio=0.85
All	Logistic regression	C=27.5, L1 ratio=0.775

Figure S4. The optimal hyperparameter and algorithms for each input feature. A: Hyperparameter optimization based on kappa scores. The results of hyperparameter optimization for “drug response” single view and a pairwise combination “cancer progression plus drug response” were shown as examples. B: Algorithms and parameters selected for each view. The best performed ML classifier and their optimal hyperparameters for each view were identified for the prediction of the cancer genes and the host genes of the tumor in HCC PDX models, respectively.

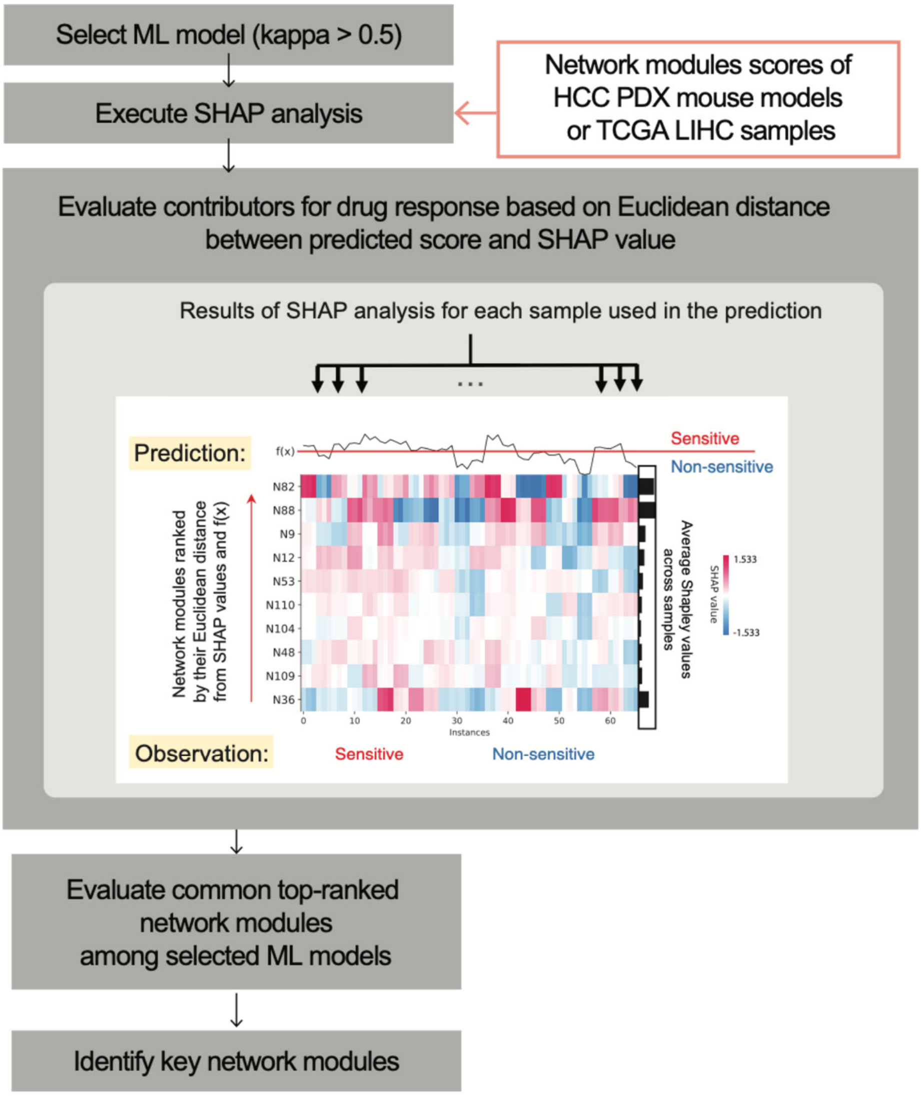

Figure S5. Scheme of SHAP score-based identification of key non-canonical network modules for lenvatinib response.

Supplementary Tables

Table S1. (attached). List of non-canonical network modules. Each sheet corresponds to the input type of non-canonical network modules ("cancer progression," "drug treatment," and "drug response"). Key genes related to angiogenesis, genes related to T-cell inflammation, and T-cell or tumor associated macrophages phenotype markers are marked.

Table S2. (attached). RNA sequencing data (TPM values) of human and mouse genes in tumors from the HCC PDX models.

Table S3. Number of predictive ML models with kappa scores of >0.5 for cancer genes or host genes in tumors from the HCC PDX models.

	DR	CP	DT	DR+CP	DR+DT	CP+DT	All (DR+CP+DT)
Cancer genes in the HCC PDX models	76	0	0	191	23	0	179
Host genes in the HCC PDX models	5	223	0	222	31	180	178

DR: drug response, CP: cancer progression, DT: drug treatment.

Table S4. (attached). Important non-canonical network modules for predicting sensitivity to lenvatinib (average ranking < 15). Related to Table 1.

Table S5. (attached). Pathway enriched analysis of the network modules in Table 1. List of member genes of non-canonical network modules and enriched terms based on the DAVID analysis. No enriched terms were identified for "drug response (baseline) N34" and "drug response (baseline) N42."

Table S6. (attached). TCGA sample identifiers for the LIHC RNA-seq dataset used in this study. List of TCGA barcode IDs corresponding to the 371 liver hepatocellular carcinoma (LIHC) tumor samples obtained from the Broad GDAC Firehose and analyzed in this study.

Table S7. The 15 HCC PDX model samples of which host genes in the tumor were qualified.

Samples	Sen/NS	Slope (genes/1,000 reads)
LI6617_1752	Sen	0.6
LI6617_1759	Sen	0.78
LI0334_3696	Sen	0.76
LI0334_3709	Sen	0.16
LI1054_8250	Sen	0.76
LI1054_8262	Sen	0.5
LI1054_8267	Sen	0.78
LI1025_4122	NS	0.44
LI1025_4126	NS	0.94
LI6662_2937	NS	0.28
LI6662_2943	NS	0.8
LI0574_7091	NS	0.7
LI6653_6028	NS	0.44
LI0050_353	NS	0.68
LI1037_3763	NS	0.66

Samples represents sample ID and replicate ID concatenated with underscore. Sen: sensitive, NS: non-sensitive.

Table S8. ANOVA/Tukey adjusted *p*-values for pairwise comparisons among seven non-canonical feature-views (cancer-gene classifiers).

Comparison	Feature 1	Feature 2	Adjusted p-value (accuracy)	Adjusted p-value (precision)	Adjusted p-value (F1 score)	Adjusted p-value (Kappa score)	Adjusted p-value (AUC score)	Stars (accuracy)	Stars (precision)	Stars (F1 score)	Star (Kappa score)	Star (AUC score)
DT-All	DT	All	0.000E+00	0.000E+00	0.000E+00	0.000E+00	0.000E+00	***	***	***	***	***
DT-CP	DT	CP	0.000E+00	0.000E+00	0.000E+00	0.000E+00	0.000E+00	***	***	***	***	***
DT-CP+DT	DT	CP+DT	0.000E+00	0.000E+00	0.000E+00	0.000E+00	0.000E+00	***	***	***	***	***
DT-DR	DT	DR	0.000E+00	0.000E+00	0.000E+00	0.000E+00	0.000E+00	***	***	***	***	***
DT-DR+CP	DT	DR+CP	0.000E+00	0.000E+00	0.000E+00	0.000E+00	0.000E+00	***	***	***	***	***
DT-DR+DT	DT	DR+DT	0.000E+00	0.000E+00	0.000E+00	0.000E+00	0.000E+00	***	***	***	***	***
DR+DT-All	DR+DT	All	0.000E+00	0.000E+00	0.000E+00	0.000E+00	0.000E+00	***	***	***	***	***
DR+DT-CP	DR+DT	CP	0.000E+00	0.000E+00	0.000E+00	0.000E+00	0.000E+00	***	***	***	***	***
DR+DT-CP+DT	DR+DT	CP+DT	0.000E+00	0.000E+00	0.000E+00	0.000E+00	0.000E+00	***	***	***	***	***
DR+DT-DR	DR+DT	DR	0.000E+00	0.000E+00	6.121E-10	0.000E+00	0.000E+00	***	***	***	***	***
DR+DT-DR+CP	DR+DT	DR+CP	0.000E+00	0.000E+00	0.000E+00	0.000E+00	0.000E+00	***	***	***	***	***
DR+CP-All	DR+CP	All	2.604E-01	9.886E-01	2.085E-04	4.858E-03	2.947E-06			***	**	***
DR+CP-CP	DR+CP	CP	0.000E+00	0.000E+00	0.000E+00	0.000E+00	0.000E+00	***	***	***	***	***
DR+CP-CP+DT	DR+CP	CP+DT	0.000E+00	0.000E+00	0.000E+00	0.000E+00	0.000E+00	***	***	***	***	***
DR+CP-DR	DR+CP	DR	0.000E+00	1.526E-07	0.000E+00	0.000E+00	0.000E+00	***	***	***	***	***
DR-All	DR	All	2.152E-08	0.000E+00	1.869E-03	3.985E-06	7.671E-05	***	***	**	***	***
DR-CP	DR	CP	0.000E+00	0.000E+00	0.000E+00	0.000E+00	0.000E+00	***	***	***	***	***
DR-CP+DT	DR	CP+DT	0.000E+00	0.000E+00	0.000E+00	0.000E+00	0.000E+00	***	***	***	***	***
CP+DT-All	CP+DT	All	0.000E+00	0.000E+00	0.000E+00	0.000E+00	0.000E+00	***	***	***	***	***
CP+DT-CP	CP+DT	CP	0.000E+00	1.558E-04	1.000E+00	4.515E-02	2.766E-01	***	***		*	
CP-All	CP	All	0.000E+00	0.000E+00	0.000E+00	0.000E+00	0.000E+00	***	***	***	***	***

Note: Tukey-adjusted p-values are denoted as: *$p < 0.05$; **$p < 0.01$; ***$p < 0.001$.

Table S9. ANOVA/Tukey adjusted p-values for pairwise comparisons among seven non-canonical feature-views (host-gene classifiers).

Comparison	Feature 1	Feature 2	Adjusted p-value (accuracy)	Adjusted p-value (precision)	Adjusted p-value (F1 score)	Adjusted p-value (Kappa score)	Adjusted p-value (AUC score)	Stars (accuracy)	Stars (precision)	Stars (F1 score)	Star (Kappa score)	Star (AUC score)
DT-All	DT	All	0.000E+00	0.000E+00	0.000E+00	0.000E+00	0.000E+00	***	***	***	***	***
DT-CP	DT	CP	0.000E+00	0.000E+00	0.000E+00	0.000E+00	0.000E+00	***	***	***	***	***
DT-CP+DT	DT	CP+DT	0.000E+00	0.000E+00	0.000E+00	0.000E+00	0.000E+00	***	***	***	***	***
DT-DR	DT	DR	0.000E+00	0.000E+00	0.000E+00	0.000E+00	0.000E+00	***	***	***	***	***
DT-DR+CP	DT	DR+CP	0.000E+00	0.000E+00	0.000E+00	0.000E+00	0.000E+00	***	***	***	***	***
DT-DR+DT	DT	DR+DT	0.000E+00	0.000E+00	3.193E-06	0.000E+00	0.000E+00	***	***	***	***	***
DR+DT-All	DR+DT	All	0.000E+00	0.000E+00	0.000E+00	0.000E+00	0.000E+00	***	***	***	***	***
DR+DT-CP	DR+DT	CP	0.000E+00	0.000E+00	0.000E+00	0.000E+00	0.000E+00	***	***	***	***	***
DR+DT-CP+DT	DR+DT	CP+DT	0.000E+00	0.000E+00	0.000E+00	0.000E+00	0.000E+00	***	***	***	***	***
DR+DT-DR	DR+DT	DR	0.000E+00	0.000E+00	0.000E+00	0.000E+00	0.000E+00	***	***	***	***	***
DR+DT-DR+CP	DR+DT	DR+CP	0.000E+00	0.000E+00	0.000E+00	0.000E+00	0.000E+00	***	***	***	***	***
DR+CP-All	DR+CP	All	3.575E-05	3.325E-03	9.618E-02	7.439E-05	1.207E-04	***	**		***	***
DR+CP-CP	DR+CP	CP	3.429E-03	1.330E-01	3.868E-07	7.055E-04	8.393E-05	**		***	***	***
DR+CP-CP+DT	DR+CP	CP+DT	9.881E-02	3.325E-06	9.924E-01	2.470E-01	4.807E-01		***			
DR+CP-DR	DR+CP	DR	0.000E+00	0.000E+00	0.000E+00	0.000E+00	0.000E+00	***	***	***	***	***
DR-All	DR	All	0.000E+00	0.000E+00	0.000E+00	0.000E+00	0.000E+00	***	***	***	***	***
DR-CP	DR	CP	0.000E+00	0.000E+00	0.000E+00	0.000E+00	0.000E+00	***	***	***	***	***
DR-CP+DT	DR	CP+DT	0.000E+00	0.000E+00	0.000E+00	0.000E+00	0.000E+00	***	***	***	***	***
CP+DT-All	CP+DT	All	3.424E-01	7.306E-01	1.183E-02	2.122E-01	1.093E-01			*		
CP+DT-CP	CP+DT	CP	1.686E-09	9.655E-02	1.596E-05	1.937E-09	1.882E-10	***		***	***	***
CP-All	CP	All	0.000E+00	8.982E-01	0.000E+00	0.000E+00	0.000E+00	***		***	***	***

Note: Tukey-adjusted p-values are denoted as: *$p < 0.05$; **$p < 0.01$; ***$p < 0.001$.

Table S10. Average ranking stability of "drug response" network modules across $\Delta T/C$ thresholds. Average ranking of "drug response" network modules in Table 1 was evaluated under $\Delta T/C$ thresholds of 10% and 12%. "Enriched Function" indicates representative functional terms derived from functional term enrichment analysis (see Table S5 for details).

Table 1 Label	Network Module	Enriched Function	Ave. Ranking (12%)	Ave. Ranking (10%)
Cancer (Class I)	Drug response (baseline) N8	Angiogenesis (S100A family)	2.50	1.13
Cancer (Class I)	Drug response (baseline) N19	NGF pathway	1.00	2.26
Cancer (Class I)	Drug response (baseline) N34	N/A	4.00	3.47
Cancer (Class I)	Drug response (baseline) N40	Wnt pathway	11.50	5.61
Cancer (Class II)	Drug response (baseline) N2	SUMOylation / transcription regulation	2.50	3.46
Cancer (Class II)	Drug response (baseline) N28	Interleukins (IL-1)	11.00	12.80
Cancer (Class II)	Drug response (baseline) N38	Angiogenesis (SEMA3A)	8.00	7.91
Cancer (Class II)	Drug response (baseline) N42	N/A	9.00	14.22
Cancer (Class II)	Drug response (non-treatment) N1	Interleukins (IL-18, IL-24)	15.50	9.67

Table S11. Reproducibility of "drug response" network modules across label-perturbation analyses. Modules listed in Table 1 are shown with their enriched functions and the frequency of appearance within the top 20 ranked features across (i) valid label-perturbation runs (kappa score > 0.5; 26 runs) and (ii) perturbation runs after excluding those without effective label changes (21 runs). "Enriched Function" indicates representative functional terms derived from functional term enrichment analysis (see Table S5 for details).

Table 1 Label	Module Name	Enriched Function	Top 20 Frequency (%)	
			Valid perturbations (κ > 0.5; n=26)	Effective label perturbations only (n= 21)
Cancer (Class I)	Drug response (baseline) N8	Angiogenesis (S100A family)	84.6%	81.0%
Cancer (Class I)	Drug response (baseline) N19	NGF pathway	100.0%	100.0%
Cancer (Class I)	Drug response (baseline) N34	N/A	80.8%	76.2%
Cancer (Class I)	Drug response (baseline) N40	Wnt pathway	84.6%	81.0%
Cancer (Class II)	Drug response (baseline) N2	SUMOylation / transcription regulation	96.2%	95.2%
Cancer (Class II)	Drug response (baseline) N28	Interleukins (IL-1)	57.7%	71.4%
Cancer (Class II)	Drug response (baseline) N38	Angiogenesis (SEMA3A)	65.4%	57.1%
Cancer (Class II)	Drug response (baseline) N42	N/A	26.9%	33.3%
Cancer (Class II)	Drug response (non-treatment) N1	Interleukins (IL-18, IL-24)	73.1%	66.7%

Table S12. Hypergeometric test results for overlap of highly ranked network modules (average rank < 15) between HCC PDX and TCGA LIHC across comparable ML input views, including raw *p*-values and FDR-adjusted *q*-values.

HCC PDX models	View	Average ranking < 15		
		p-value	q-value	q < 0.05
Cancer cells	DR	0.0008	0.0019	*
Cancer cells	DR+CP	0.1743	0.1743	
Cancer cells	All	0.0153	0.0255	*
Host cells	DR+CP	0.0002	0.0011	*
Host cells	All	0.1125	0.1406	
