# Supplementary material for: Leveraging Network-Based Transcriptome Analysis from Mouse Tumor Models and Explainable Artificial Intelligence to Advance the Understanding of the Antitumor Activity of Lenvatinib
Source: Cancers (Basel). 2026 Mar 25;18(7):1067. doi: 10.3390/cancers18071067 (PMC13072315; doi:10.3390/cancers18071067)
Supplement: Supplementary file 1 [file cancers-18-01067-s001.zip › Eisai_TME_LEN-paper_TableS1.pdf]

Supplementary Table 1A. cancer progression network modules

Network module	Member genes
Cancer progression (4T1) N1	ABCB8, AIFM1, ARL2BP, BOK, CRABP1, DCTPP1, ESD, GRB10, H1-0, HSH2D, HSPA1A, KBTBD4, L3MBTL2, MDH1, MORN2, MPP3, NFU1, NOA1, PLPP6, RAB31, STAC3, ZPR1
Cancer progression (4T1) N2	AARS1, ASNS, BUB1, BUB1B, CCNB1, CDC20, CENPC, CENPE, COMMD4, DCPS, DSN1, ESPL1, FBXO5, KNL1, MAD2L1, MIS12, NSL1, NUF2, PMF1, PTTG1, SPC24, SPC25
Cancer progression (4T1) N3	B3GALNT1, CCNA2, CDC45, CDC6, CDC7, CDT1, DBF4, GINS1, GINS2, GINS3, GINS4, GMNN, HDAC11, IMPDH1, IMPDH2, MCM3, MCM4, MCM5, ORC1, ORC6
Cancer progression (4T1) N4	AASDHPPT, ASAH1, CAMK1D, FASN, GMFG, LGALS3BP, NDUFAB1, NT5C3A, PRR12, PSD, RAPGEF5, TMEM253, TNFRSF13B, TNFSF13, TNFSF13B, VANGL1
Cancer progression (4T1) N5	ACKR3, ADAMTS5, ATF6, BMF, CCL11, CCL24, CCL3L1, CCL7, CCR2, CXCL10, CXCL12, CXCL9[§], DPP4, MMP2[*], NNMT, TIMP3[*]
Cancer progression (4T1) N6	CYC1, ENO2, HK1, HOOK1, NDUFA13, NDUFAF4, NDUFS5, NDUFS7, NDUFS8, SIGIRR, SSNA1, ST3GAL2, TBC1D7, TIMMDC1, UBE2C
Cancer progression (4T1) N7	AK5, AKR1B1, ITGB1BP2, NUCKS1, PDCD2L, PLOD2, PUM3, RNF125, SORD, TALDO1, TKT, TRIM46, UGDH, XPO5
Cancer progression (4T1) N8	ACTA1, CAPG, CNN1, DNASE1, FGD4, MAP1A, NCAPD2, NCAPD3, NCAPG, NCAPG2, NCAPH, SMC2, SMC4
Cancer progression (4T1) N9	CD36[¶], COL1A1, COL1A2, COL5A1, COL5A3, CXCL6, CXCR2, DCN, FAM20B, LCN2, MMP9[*], SDC3, TGFBI
Cancer progression (4T1) N10	ELN, FBLN2, FBLN5, FBN1, FBN2, LOX, LOXL1, MAGEH1, MFAP2, MMP12, MOAP1, NID1
Cancer progression (4T1) N11	ACAP2, CBR3, DENND1A, DENND1B, GNPAT, IDH3A, IDH3B, IDH3G, RAB35, SUCLA2, SUCLG1, SUCLG2
Cancer progression (4T1) N12	BLM, FANCA, FANCM, FEN1, MDC1, MRE11, NBN, RMI1, RMI2, RPA3, TOP3A
Cancer progression (4T1) N13	CACTIN, HACD3, HDLBP, HMGB2, HOXB6, LST1, PKNOX1, SAMM50, ZNF428, ZNF622, ZNF668
Cancer progression (4T1) N14	BRIP1, CD200, CHEK1, CLSPN, DTL, HUS1, PIGM, RAD1, RAD9A, RHNO1, TOPBP1
Cancer progression (4T1) N15	CMTM3, COL3A1, FST, GDF11, INHBA, MEST, MPZL2, PCOLCE, SNX7, SPARC, ZNF579
Cancer progression (4T1) N16	DDX54, F2RL1, PLXND1, SEMA3E, SEMA4A, TMED10, TMED2, TMED4, TMED7, TMED9
Cancer progression (4T1) N17	CBR1, CD27[§], ERCC8, GCOM1, MYZAP, PRKAB1, PRKAG2, SIVA1, UQCRQ, WDR89
Cancer progression (4T1) N18	CDC42BPB, CLASP2, DBI, GOLPH3, MYO18A, NIPSNAP1, NIPSNAP2, PLEC, PPP1R3B, PYGM
Cancer progression (4T1) N19	ABCC1, DDOST, FAM162A, MAGT1, NXT1, RPN2, SSR4, STT3B, VDAC2, VDAC3
Cancer progression (4T1) N20	COL6A2, COX6A2, MRPL1, MRPL10, MRPL12, MRPS18B, MRPS22, MRPS26, SNX21, TMEM51
Cancer progression (4T1) N21	AHCTF1, HOXC10, NUP107, NUP133, NUP160, NUP205, NUP37, NUP85, NUP93
Cancer progression (4T1) N22	ARSA, CETN2, CETN3, POC5, PPIC, SGSM1, SUMF1, UBAP1, USP44
Cancer progression (4T1) N23	E2F3, E2F4, E2F5, E2F6, KDM5C, LIN54, LIN9, MYBL2, TFDP1
Cancer progression (4T1) N24	AKAP1, AKAP5, PRKAR2B, TAF11, TAF12, TAF13, TAF3, TAF5
Cancer progression (4T1) N25	ARF6, CNKSR1, CYTH2, CYTH3, ECT2, KIF23, RACGAP1, SPAG9
Cancer progression (4T1) N26	CCT8, DKC1, GAR1, LYPLA2, NAF1, NHP2, NOP10, WRAP53
Cancer progression (4T1) N27	EFS, GLE1, MAP4K3, NUP155, NUP42, SEMA6A, SH3RF1, SORBS2
Cancer progression (4T1) N28	ANGPT1[*], ANGPT4, ANGPTL1, ANGPTL2, ANGPTL4[*], PTPRG, TEK[*], TIE1[*]
Cancer progression (4T1) N29	EIF2AK1, EIF2B1, EIF2B3, EIF2B4, EIF2B5, EIF2S1, EIF2S2, EIF2S3
Cancer progression (4T1) N30	CEP152, CEP192, EPB41L3, PLK4, SMYD2, STIL, TENT5C, UNC13A
Cancer progression (4T1) N31	C1R, C1S, C2, C3, C3AR1, C4B, CD46, MASP1
Cancer progression (4T1) N32	ABCC5, AK2, CCDC141, NME1, NME2, PID1, RRAD
Cancer progression (4T1) N33	DLAT, DLD, HIC2, ISOC2, OGDH, PDK2, PDK3
Cancer progression (4T1) N34	IGF1, IGFBP3, IGFBP4, IGFBP5, IGFBP6, IGFBP7, PNO1
Cancer progression (4T1) N35	ADM, CALCRL[*], CORT, GHRL, GPR39, RAMP2[*], RAMP3
Cancer progression (4T1) N36	BAZ1A, CHRAC1, DR1, POLE, POLE2, POLE3, TBX15
Cancer progression (4T1) N37	FZD1, FZD2, FZD6, KIAA1522, RYK, SFRP1, WNT5A
Cancer progression (4T1) N38	EID3, NSMCE1, NSMCE2, NSMCE3, NSMCE4A, SMC5, SMC6
Cancer progression (4T1) N39	ARL6, BBS1, BBS10, BBS5, BBS7, MKKS, TTC8
Cancer progression (4T1) N40	COX4I2, CYB5A, CYCS, GSTK1, IFT57, PIP4K2A, TIA1
Cancer progression (4T1) N41	ARPC1A, ARPC1B, ARPC2, ARPC4, ARPC5, ARPC5L, TTLL3
Cancer progression (4T1) N42	CRCP, POLR2F, POLR2H, POLR3B, POLR3K, TSR1
Cancer progression (4T1) N43	PODXL, SELENOP, SELL, SELP, SELPLG, VCAN
Cancer progression (4T1) N44	CHTF18, DSCC1, RFC2, RFC3, RFC4, RFC5
Cancer progression (4T1) N45	FAN1, FANCD2, FANCI, PMS1, PMS2, USP1
Cancer progression (4T1) N46	CAB39, DES, IPO5, NEB, RSL1D1, STRADB
Cancer progression (4T1) N47	CMTM4, MRPL13, MRPL2, MRPL22, MRPS7, TUFM
Cancer progression (4T1) N48	APMAP, JPT2, LOXL3, OVGP1, RASA4, SLC25A20
Cancer progression (4T1) N49	CMSS1, GYG1, GYS1, PDGFB[*], S1PR1, TRIM7
Cancer progression (4T1) N50	DMTN, DYNC1LI1, DYNC1LI2, DYNLT3, PROCR, PTOV1
Cancer progression (4T1) N51	DEGS1, GRAP, LAT, NAGPA, SHB, ZHX2
Cancer progression (4T1) N52	CSF1R, CSF2, CSF2RA, CSF3, IL34, SOCS1
Cancer progression (4T1) N53	EBNA1BP2, IFI16, MKI67[§], NIFK, RPF2, RRS1

Network module	Member genes
Cancer progression (4T1) N54	CLDN12, MIOS, SEC13, SEC16B, SESN2, WDR24
Cancer progression (4T1) N55	MZT1, TUBG1, TUBGCP2, TUBGCP3, TUBGCP5, TUBGCP6
Cancer progression (4T1) N56	GFRA1, NCAM1, ROBO1, ROBO4[*], SLIT2, SLIT3
Cancer progression (B16F10) N1	CCL11, CCL2, CCL3, CCL4, CCL7, CCL8, CCR2, CCR5, CD36[¶], CEBPA, CIITA, COL1A1, COL1A2, COL5A1, COL5A3, CXCL6, DCN, FAM20B, MMP13, MMP2[*], MMP3, MMP9[*], RFX5, RFXANK, SDC3, SPI1, TGFBI, TIMP1
Cancer progression (B16F10) N2	ADAMTS1, BGN, CD34[*], ESAM[*], PODXL, PODXL2, SELENOP, SELL, SELPLG, TLR1, VCAN
Cancer progression (B16F10) N4	CABP1, ITPR2, ITPR3, ORAI1, ORAI3, STIM1, TRPC1
Cancer progression (B16F10) N5	ICAM1[*], ICAM2[*], ITGAL, ITGAM[§¶], ITGAX[§], ITGB2
Cancer progression (B16F10) N6	BAMBI, FSTL3, INHBA, SELENOF, TGFB2, TGFBR3
Cancer progression (B16F10) N7	BST1, CD14[¶], FSTL1, GJB2, LBP
Cancer progression (BNL) N1	AKAP12, ANKRD1, ASPH, C1QTNF9, CACNA1S, CSRP3, DES, FHL1, KLHL41, MYBPC1, MYOM1, MYOM2, MYOZ1, NEB, NRAP, OBSL1, PALLD, PDLIM3, RYR1, TCAP, TRDN, TXNIP, ZBTB40
Cancer progression (BNL) N2	ADAM12, HFE, IGF1, IGFBP2, IGFBP3, IGFBP4, IGFBP5, TF
Cancer progression (BNL) N3	ACTA1, DMD, DNASE1, DTNA, FGD4, PGM5
Cancer progression (BNL) N4	CCL22, CRYAA, CRYAB, GCLC, HERC3
Cancer progression (BNL) N5	ACKR4, CCL11, CCL24, CCL7, CCR2
Cancer progression (CT26) N1	CCL11, CCL22, CCL24, CCL3L1, CCL8, CCR2, CXCL10, CXCR3, DPP4, GCLC, HERC3
Cancer progression (CT26) N2	CD274[§], CD28[§], CD80[§], CD86[§¶], CTLA4[§], GRAP, ITK, LAT, PDCD1[§¶], PDCD1LG2[§¶]
Cancer progression (CT26) N3	CLCF1, CRLF1, LIF, LIFR, OSM, OSMR
Cancer progression (CT26) N4	ESAM[*], SELE, SELENOP, SELP, SELPLG, VWF
Cancer progression (CT26) N5	ARL4D, CDH1, CNPY4, NDRG1, PIP5K1C, PTPRF
Cancer progression (CT26) N6	ACE, AGT, ANPEP, BDKRB2, CTSG, PRCP
Cancer progression (CT26) N7	TNNC1, TNNC2, TNNI2, TNNI3, TNNT2, TNNT3
Cancer progression (CT26) N8	CD244, CD48, IL18, IL18BP, IL18R1, IL1RL2
Cancer progression (EMT6) N1	ADAMTS5, CCL2, CCL7, CCL8, CCR2, MMP2[*], MMP3, TIMP1, TIMP3[*]
Cancer progression (EMT6) N2	CD34[*], ESAM[*], PODXL, PODXL2, SELENOP, SELL, SELP, SELPLG, SERPING1
Cancer progression (EMT6) N3	ACTN2, AOC3, COL12A1, GAS7, MYBPC1, MYBPC2, PCDH12, TRIP11
Cancer progression (EMT6) N5	ASPH, F10, PDLIM3, RYR1, TFPI, TRDN
Cancer progression (EMT6) N6	ICAM1[*], ICAM2[*], IL2RA, IL2RB, ITGAL, ITGAX[§]
Cancer progression (EMT6) N7	IL1R1, IL1R2, IL1RAP, IL1RL2, IL1RN
Cancer progression (LL2) N1	CD36[¶], CIITA, COL1A1, COL1A2, COL5A1, COL5A3, MMP9[*], RFX5, RFXANK, RFXAP, TGFBI
Cancer progression (LL2) N2	ADRB2, AKAP12, FHL1, GNA15, HES1, SLC52A2, ZBTB40
Cancer progression (LL2) N3	ACAP1, F3, GPR162, HFE, TF, TFRC
Cancer progression (LL2) N4	DEGS1, GRAP, ITK, LAT, NAGPA, ZHX2
Cancer progression (MBT2) N1	C1S, C2, C4B, ESAM[*], LPAR1, LPAR2, PODXL, SELENOP, SELL, SELP, SELPLG, SERPING1, SLC9A3R2[*]
Cancer progression (MBT2) N2	CCL11, CCL22, CCL24, CCNQ, CCR2, CXCL12, CXCL9[§], CXCR3, DPP4, HERC3, PTPN5, TMEM51
Cancer progression (MBT2) N3	CIITA, CYBA, CYBB, IL1B, IL1R2, IL1RL2, IL1RN, IRF8, NCF2, NCF4, RFX5
Cancer progression (MBT2) N4	CD36[¶], COL1A1, COL1A2, COL5A1, COL5A3, DCN, FLT4, ITGA2B, TGFBI, THBS1
Cancer progression (MBT2) N6	ITGA7, ITGA8, LAMA2, NPNT[*], NT5E, TNC
Cancer progression (MBT2) N7	CSF1R, CSF2, CSF2RA, CSF2RB, CSF3R, IL34
Cancer progression (MBT2) N9	ADAMTS5, MMP2[*], MMP25, MMP3, TIMP3[*]
Cancer progression (MBT2) N10	IGF1, IGFBP5, IGFBP6, IGFBP7, PAPPA2
Cancer progression (MBT2) N12	ADM, CALCRL[*], IAPP, RAMP2[*], RAMP3
Cancer progression (MBT2) N13	EDNRB, GDNF, GFRA1, GFRA2, NCAM1
Cancer progression (Renca) N1	CD36[¶], COL1A1, COL1A2, COL5A1, COL5A3, DCN, FAM20B, ITGA11, TGFBI
Cancer progression (Renca) N2	ASPH, HOMER1, IRAG1, MYOM2, PALLD, PDLIM3, PRKG1, RYR1, TRDN
Cancer progression (Renca) N4	ADM, ADM2, CALCRL[*], CORT, RAMP1, RAMP3
Cancer progression (Renca) N6	CIITA, IL1B, IL1R2, IL1RL2, IL1RN, IRF4
Cancer progression (Renca) N7	CNTF, LIF, LIFR, OSM, OSMR
Cancer progression (Renca) N8	SEPTIN11, SEPTIN3, SEPTIN5, SEPTIN6, SEPTIN7

*: angiogenesis, §: T-cell activation related (T-cell phenotypes markers and TIS), ¶: TAM phenotypes markers

Supplementary Table 1B. drug treatment network modules

Network module	Member genes
Drug treatment (B16F10) N1	CASP1, CEBPA, CIITA, CSF1, CSF1R, CYBA, CYBB, IL1B, IL1R1, IL1RN, IL33, IL34, IRF1, IRF8, NCF1, NCF4, RFX5, SOCS1, SPI1
Drug treatment (B16F10) N2	BGN, BST1, CD14[¶], CD34[*], ESAM[*], FSTL1, GJB2, LBP, PODXL, PODXL2, SELL, SELPLG, TLR1, TLR2, VCAN
Drug treatment (B16F10) N3	C3, C3AR1, CFB, CFD, CFP, ICAM1[*], ICAM2[*], IFITM1, ITGAL, ITGAM[§¶], ITGAX[§], ITGB2, PTX3
Drug treatment (B16F10) N4	CCL11, CCL2, CCL7, CCL8, CCR2, COL1A2, CXCL6, MMP13, MMP2[*], MMP3, MMP9[*], TIMP1
Drug treatment (B16F10) N6	ITPR2, ITPR3, ORAI1, ORAI3, STIM1, TRPC1
Drug treatment (B16F10) N7	CD36[¶], COL1A1, DCN, FAM20B, TGFBI
Drug treatment (BNL) N1	AKAP12, ANK1, ANKRD1, AOC3, C1QTNF9, CACNA1S, CAPN3, CCL22, CRYAB, CSRP3, DES, DSP, ENO3, FHL1, GCLC, GCLM, HERC3, IGF2, IGFBP2, IGFBP5, ITPR3, KLHL41, LDB2, MYBPC1, MYBPC2, MYBPH, MYL1, MYOM1, MYOM2, MYOZ1, NEB, NRAP, OBSCN, ORAI1, ORAI3, PCDH12, PDLIM3, PKP2, PREPL, RYR1, SPTB, STIM1, TCAP, TRDN, TRPC1
Drug treatment (BNL) N2	CD34[*], ESAM[*], EZR, PODXL, PODXL2, SELL, SELPLG, SLC12A2, SLC9A3R2[*]
Drug treatment (CT26) N1	ADAM12, F13A1, HFE, IGF1, IGFBP3, IGFBP4, IGFBP5, IGFBP6, IGFBP7, ITGA9, LAPTM4A, TF, TUBB4A
Drug treatment (CT26) N2	CCL22, CRYAB, GCLC, HERC3, HSPB2, HSPB8
Drug treatment (CT26) N3	CD300C, FST, FSTL3, GDF11, INHBA, MEST
Drug treatment (CT26) N4	HTR2B, HTR2C, MPDZ, PLEKHA1, PLEKHA2, TPH1
Drug treatment (CT26) N5	CD274[§], CD80[§], CTLA4[§], PDCD1[§¶], PDCD1LG2[§¶]
Drug treatment (CT26) N6	ASPH, F10, F8, MGST3, PROS1
Drug treatment (CT26) N7	EIF5B, GATA4, LEF1, ZBTB3, ZNF556
Drug treatment (Hepa) N1	F3, F7, FANCB, GPR162, LRP11, LTF, POGLUT1
Drug treatment (Hepa) N2	ELN, FBLN2, FBN1, LOX, MAGEH1, MFAP2, MMP12
Drug treatment (Hepa) N3	IRAG1, MYOZ2, PALLD, PDLIM3, PRKG1, RYR1
Drug treatment (Hepa) N4	HNF4G, TNNC1, TNNC2, TNNI2, TNNI3, TNNT3
Drug treatment (Hepa) N5	IL1A, IL1B, IL1R2, IL1RAP, IL1RL2, IL1RN
Drug treatment (Hepa) N6	ITPR2, ORAI1, ORAI3, STIM1, TRPC1, TRPC3
Drug treatment (Hepa) N7	CYBA, CYBB, NCF1, NCF2, NCF4
Drug treatment (Hepa) N8	MLPH, MYL6B, MYO5A, SPA17, SPIRE2
Drug treatment (Hepa) N9	CD34[*], PODXL2, SELL, SELP, SELPLG
Drug treatment (KLN205) N1	ARHGDIB, CIITA, CYBA, CYBB, IRF8, NCF2, NCF4, RAC2, RFX5, RFXANK, RHOH, SPI1
Drug treatment (KLN205) N2	ELN, FBLN5, FBN1, FBN2, LOX, LOXL1, MFAP2, MMP12
Drug treatment (KLN205) N3	CISH, CYP3A5, ETV5, FOXA1, OASL, RDH13, SEZ6L2, UNG
Drug treatment (KLN205) N4	BUB1, BUB1B, CENPE, KNL1, NSL1, NUF2, SPC24, SPC25
Drug treatment (KLN205) N5	CD34[*], PODXL, PODXL2, SELENOP, SELL, SLC9A3R2[*], VWF
Drug treatment (KLN205) N6	CNTF, CNTFR, CTF1, IL6R, LIFR, OSM, OSMR
Drug treatment (KLN205) N7	GFRA1, NCAM1, NRTN, ROBO1, ROBO4[*], SLIT2, SLIT3
Drug treatment (KLN205) N8	ASAH1, CAMK1D, GMFG, PSD, TNFRSF13B, TNFSF13
Drug treatment (KLN205) N9	ADM2, CALCB, CALCRL[*], RAMP1, RAMP2[*], RAMP3
Drug treatment (KLN205) N10	CNN1, NCAPD2, NCAPG, NCAPH, SMC4
Drug treatment (KLN205) N11	SEMA6D, SIRPB1, TREM1, TREM2, TYROBP
Drug treatment (KLN205) N12	ANGPT4, ANGPTL1, ANGPTL2, TEK[*], TIE1[*]
Drug treatment (KLN205) N13	FZD2, KIAA1522, SFRP1, TMEM80, WNT4
Drug treatment (LL2) N1	HFE, IGF1, IGFBP3, IGFBP6, IGFBP7, SH3BP2, TF, TFRC, TIMELESS, TIPIN
Drug treatment (LL2) N2	ACE, AGTR2, CCL2, CCL8, MMP2[*], MMP3, TIMP2[*], TIMP3[*]
Drug treatment (LL2) N3	EFNB3, EPHB3, EPHB4[*], GBE1, TBC1D2B, TRDMT1
Drug treatment (LL2) N4	C1QA, C1QB, C1QC, CR1, PTX3, SLC25A47
Drug treatment (LL2) N5	CD36[¶], COL5A1, COL5A3, SDC3, THBS1
Drug treatment (LL2) N7	CSF1, CSF1R, CSF3, CSF3R, IL34
Drug treatment (LL2) N8	ARL6, BBS1, BBS2, BBS5, BBS9
Drug treatment (MC38) N1	CCL21, CCL3L1, CCR7[§], CXCL10, CXCL12, CXCL9[§], CXCR3, DPP4
Drug treatment (MC38) N2	EFNB2, EFNB3, EPHB2, EPHB3, GBE1, PECAM1[*], RHBDL2
Drug treatment (MC38) N3	CD36[¶], COL1A1, COL1A2, DCN, FAM20B, ITGA2B
Drug treatment (MC38) N6	ADAMTS5, MMP2[*], MMP3, TIMP2[*], TIMP3[*]
Drug treatment (MC38) N8	CD34[*], PODXL, SELENOP, SELL, SELPLG
Drug treatment (MC38) N9	BGN, CD180, LY86, LY96, TLR1
Drug treatment (RAG) N1	ADAM12, F3, F7, FANCB, GPR162, HFE, IGF1, IGFBP3, IGFBP6, IGFBP7, POGLUT1, TF, TFRC, TIMELESS
Drug treatment (RAG) N2	CD48, IL18, IL18BP, IL18R1, IL18RAP, IL1A, IL1R2, IL1RL2, IL1RN

Network module	Member genes
Drug treatment (Renca) N1	ADAM12, CSF1R, CSF2, CSF2RA, F13A1, FLT1*, HFE, IGFBP3, IL34, ITGA9, NRP1*, PGF, SEMA3F, SOCS1, TF, TFR2, TIMELESS, TIPIN, VEGFD
Drug treatment (Renca) N2	ATXN7L2, ITGA6, LAMA2, LAMA4, LAMB1, LAMB3, MDK, PPP2R3A, THBS2
Drug treatment (Renca) N3	AFG3L2, ARHGDIB, CYBB, NCF1, NCF2, NCF4, RAC2, RHOH
Drug treatment (Renca) N4	CLCF1, CTF1, LIF, LIFR, OSM, OSMR
Drug treatment (Renca) N5	ASPH, MYOM2, PDLIM3, RYR1, TRDN
Drug treatment (Renca) N7	CISH, DHRS7, ETV5, OASL, SEZ6L2
Drug treatment (Renca) N8	BMF, CXCL9§, NNMT, PTPN5, TMEM51

*: angiogenesis, §: T-cell activation related (T-cell phenotypes markers and TIS), ¶: TAM phenotypes markers

Supplementary Table 1C. drug response network modules

Network module	Member genes
Drug response (baseline) N1	CTHRC1, FZD1, FZD10, FZD6, FZD8, KIAA1522, NOTUM, SFRP1, TMEM80, WNT16, WNT4, WNT5A, WNT7A, WNT7B
Drug response (baseline) N2	ALB, CENPT, CENPW, DHX29, FCGRT, HNF4A, LBH, NR3C2, NR5A1, NR5A2, PAWR, PISD, PPARGC1A, PROX1
Drug response (baseline) N3	DLX3, EMX2, HOXA11, HOXA13, HOXA9, HOXC11, HOXD13, IRF6, MEIS1, MEIS2, PBX1, TLX2, TRMT10B
Drug response (baseline) N4	BAHCC1, BCAM, CLCA1, CLSTN1, COL17A1, DUSP18, ITGB4, LAMA5, LAMB3, LAMC2, SLK
Drug response (baseline) N5	EFNA5, EFNB2, EPHA3, EPHA4, EPHA7, EPHB2, EPHB3, EPHB4*, GBE1, PGD, RHBDL2
Drug response (baseline) N6	ADM, ADM2, CALCB, CALCRL*, CORT, GPR182, MRGPRX2, RAMP2*, RAMP3, SSTR3
Drug response (baseline) N7	ACKR1, CCL17, CCL8, CELF1, CXCL6, CXCR2, MOB3B, PLEKHJ1, PMAIP1, PPBP
Drug response (baseline) N8	ADTRP, ALG2, ANXA11, AREG, CCN3, S100A2, S100A4, S100A6, S100B, STK38
Drug response (baseline) N9	MLPH, RAB27A, RAB27B, SPIRE2, SYTL1, SYTL2, SYTL3, SYTL5, UNC13D
Drug response (baseline) N10	ACE, ADAMTS5, AGT, AGTR2, ANPEP, BDKRB2, MMP3, PRCP, TIMP3*
Drug response (baseline) N11	ARTN, GFRA1, NCAM1, RET, ROBO1, ROBO3, ROBO4*, SLIT2, SRGAP2
Drug response (baseline) N12	CLCF1, CNTFR, CRLF1, CTF1, LIF, LIFR, OSM, OSMR
Drug response (baseline) N13	F3, F7, FANCB, GPR162, LRP11, LTF, POGLUT1, TFR2
Drug response (baseline) N14	ADORA2B, DRAXIN, METTL1, NTN1, TPD52L2, UNC5A, UNC5B, UNC5C
Drug response (baseline) N15	GNAO1, GNAT1, HGFAC, IL6, IL6R, RGS16, SPINT1, ST14
Drug response (baseline) N16	FOXJ1, NMRAL1, RPUSD1, TRMT6, TRMT61A, TWF2, VPS36
Drug response (baseline) N17	IGF1, IGFALS, IGFBP1, IGFBP2, IGFBP5, IGFBP6, IGFBP7
Drug response (baseline) N18	BEX1, CDH1, OMP, PAX2, PLEKHA7, PTPRF, PTPRM*
Drug response (baseline) N19	BDNF, DRD4, NGF, NLN, NTF4, NTS, SORT1
Drug response (baseline) N20	AFP, ANKFY1, EHD1, EHD3, EHD4*, MICALL1
Drug response (baseline) N21	ESD, GLS2, GRHPR, KIF1B, MDH1, PGM1
Drug response (baseline) N22	ANK1, CABP1, CACNA1C, ITPR3, OBSCN, TRPC6
Drug response (baseline) N23	BCL11A, FAM72A, MAP6, MPPED2, NR2F1, YME1L1
Drug response (baseline) N24	BAMBI, FSTL3, INHBA, SELENOF, TGFB2, TGFBR3
Drug response (baseline) N25	IL13, IL13RA1, IL4, IL4R, NAGS, TMEM39B
Drug response (baseline) N26	APOA1, APOC1, GRK5, LCAT, PLTP*, SNCA
Drug response (baseline) N27	BST1, C4BPA, CD14¶, FSTL1, GJB2, LBP
Drug response (baseline) N28	IL1B, IL1R1, IL1R2, IL1RAP, IL1RL2, IL1RN
Drug response (baseline) N29	BLVRB, GLUD1, HPX, ORM1, SEC62
Drug response (baseline) N30	BPGM, EHD2, NEK3, NMNAT1, TAGLN2
Drug response (baseline) N31	FLT4, LYVE1, PDGFC, VEGFC, VEGFD
Drug response (baseline) N32	CAMLG, TNFRSF13B, TNFRSF13C, TNFRSF17, TNFSF13B
Drug response (baseline) N34	CDA, FAM178B, PTGER3, ZIC4, ZNF598
Drug response (baseline) N35	BOC, CDON, DHH, HHIP, IHH
Drug response (baseline) N36	ADAM11, ARHGEF40, EIF2B3, EIF2B4, STC2
Drug response (baseline) N37	ADGRB2, ADGRL1, ADGRL2, SHANK1, SHANK2
Drug response (baseline) N38	CEMIP, PLXNA2, PLXNA4, SEMA3A, SEMA6A
Drug response (baseline) N39	DNAAF10, FKBP6, KSR2, OSBP, PIWIL4
Drug response (baseline) N40	LGR4, LGR5, LGR6, RSPO2, RSPO4
Drug response (baseline) N41	BSCL2, EMP1, NSG1, PMP22, SMIM3
Drug response (baseline) N42	COX4I2, CYB5A, CYCS, TIA1, UGT1A4
Drug response (baseline) N43	F10, MGST3, PROZ, SERPINA10, SERPINC1
Drug response (baseline) N44	SERP1, TEX264, TMEM100, TMEM79, TMPRSS4
Drug response (non-treatment) N1	ACE, ADAMTS5, AGTR2, BDKRB2, MMP2*, MMP3, TIMP1, TIMP3*
Drug response (non-treatment) N3	CLCA1, CLCA2, COL17A1, ITGB4, LAMB3, LAMC2
Drug response (non-treatment) N5	ELN, FBLN5, LOX, LOXL1, MFAP2, MMP12

*: angiogenesis, §: T-cell activation related (T-cell phenotypes markers and TIS), ¶: TAM phenotypes markers
