# Supplementary material for: Leveraging Network-Based Transcriptome Analysis from Mouse Tumor Models and Explainable Artificial Intelligence to Advance the Understanding of the Antitumor Activity of Lenvatinib
Source: Cancers (Basel). 2026 Mar 25;18(7):1067. doi: 10.3390/cancers18071067 (PMC13072315; doi:10.3390/cancers18071067)
Supplement: Supplementary file 1 [file cancers-18-01067-s001.zip › Eisai_TME_LEN-paper_TableS4_tracked.pdf]

**Table S4**. Important non-canonical network modules for predicting sensitivity to lenvatinib (average ranking < 15). Related to Table 1.

This table summarizes non-canonical network modules that achieved an average feature-ranking score of <15 in machine-learning models trained on HCC PDX data (cancer-cell or host-cell gene expression) and/or evaluated in TCGA LIHC patient transcriptomic data. Modules are categorized into Class I (average rank <6; highlighted in red) and Class II (6 ≤ average rank <15), based on their relative importance within each analysis context. This table serves as a comprehensive candidate list from which modules consistently identified across independent datasets were selected for inclusion in Table 1.

| Network module name | PDX-TCGA overlap | | Cancer cells in HCC PDX models | | | Host cells in HCC PDX models | | | | TCGA LIHC patients | | |
|---|---|---|---|---|---|---|---|---|---|---|---|---|
| | PDX Cell Type | Class | DR | DR+CP | All | CP | DR+CP | CP+DT | All | DR | DR+CP | All |
| Drug response (baseline) N2 | Cancer | II | 3.46 | 9.33 | 9.96 | | | | | 8.14 | | |
| Drug response (baseline) N8 | Cancer | I | 1.13 | | | | | | | 1.04 | 1.47 | 1.33 |
| Drug response (baseline) N19 | Cancer | I | 2.26 | 4.95 | 4.8 | | | | | 3.08 | | |
| Drug response (baseline) N28 | Cancer | II | 12.8 | | | | | | | 12.29 | | |
| Drug response (baseline) N34 | Cancer | I | 3.47 | 3.39 | 3.36 | | 9.53 | | | 4.53 | | |
| Drug response (baseline) N38 | Cancer | II | 7.91 | | | | | | | 10.57 | | |
| Drug response (baseline) N40 | Cancer | I | 5.61 | | | | | | | 3.63 | 5.86 | 9.81 |
| Drug response (baseline) N42 | Cancer | II | 14.22 | 6.36 | 7.2 | | | | | 3.23 | 4.11 | 3.38 |
| Drug response (non-treatment) N1 | Cancer | II | 9.67 | 10.13 | 11.88 | | | | | 7.09 | | |
| Drug response (baseline) N3 | | | 14.63 | | | | | | | | | |
| Drug response (baseline) N5 | | | 10.86 | | | | | | | | | |
| Drug response (baseline) N16 | | | | 11.42 | | | | | | | | |
| Drug response (baseline) N23 | | | 11.24 | | | | | | | | | |
| Drug response (baseline) N44 | | | 11.64 | | | | | | | | | |
| Drug response (non-treatment) N3 | | | 8.83 | | | | | | | | | |
| Drug response (baseline) N4 | | | | | | | | | | 13.3 | | |
| Drug response (baseline) N32 | | | | | | | | | | 8.24 | | |
| Drug response (baseline) N37 | | | | | | | | | | 11.78 | | |
| Drug response (baseline) N39 | | | | | | | | | | 12.57 | | |
| Cancer Progression (4T1) N17 | Cancer | I | | 1.3 | 1.37 | | | | | | 2.04 | 2.55 |
| Cancer Progression (4T1) N18 | Host | II | | | | 2.22 | 3.83 | 2.12 | 3.79 | | 13.22 | |
| Cancer Progression (4T1) N32 | Host | II | | | | 10.51 | 11.23 | 12.17 | 9.13 | | 13.35 | |
| Cancer Progression (4T1) N50 | Host | II | | | | | 14.38 | | 13.75 | | 11.03 | 11.82 |
| Cancer Progression (BNL) N3 | Host | II | | | | 1.14 | 1.52 | 1.13 | 1.22 | | 8.87 | 5.16 |
| Cancer Progression (EMT6) N5 | Host | II | | | | | 3.17 | | 4.56 | | 10.39 | |
| Cancer Progression (4T1) N7 | | | | 14.51 | 11.11 | | | | | | | |
| Cancer Progression (4T1) N11 | | | | 9.42 | 10.31 | | | | | | | |
| Cancer Progression (4T1) N48 | | | | 9.27 | 7.35 | | 9.25 | | 10.38 | | | |
| Cancer Progression (CT26) N6 | | | | | | 6.33 | 14.04 | 7.22 | 10.44 | | | |
| Cancer Progression (MBT2) N2 | | | | | | 14.65 | 8.55 | | 8.84 | | | |
| Cancer Progression (Renca) N2 | | | | | | | 2.42 | | 3.1 | | | |
| Cancer Progression (4T1) N4 | | | | | | | | | | | 9.28 | 12.18 |
| Cancer Progression (4T1) N23 | | | | | | | | | | | 14.41 | 13,88 |
| Cancer Progression (4T1) N54 | | | | | | | | | | | | 14.85 |
| Drug treatment (BNL) N2 | | | | | 8.15 | | | | | | | 7.59 |
| Drug treatment (KLN205) N9 | | | | | | | | | 14.14 | | | |
| Drug treatment (KLN205) N11 | | | | | | | | | | | | 10.48 |

DR: drug response, CP: cancer progression, DT: drug treatment.
